# Thermal niche estimators and the capability of poor dispersal species to cope with climate change

**DOI:** 10.1038/srep23381

**Published:** 2016-03-17

**Authors:** David Sánchez-Fernández, Valeria Rizzo, Alexandra Cieslak, Arnaud Faille, Javier Fresneda, Ignacio Ribera

**Affiliations:** 1 Institute of Evolutionary Biology (CSIC-Universitat Pompeu Fabra), Barcelona, Spain; 2 Instituto de Ciencias Ambientales, Universidad de Castilla-La Mancha, Toledo, Spain; 3 Zoologische Staatsammlung, Muenchhausenstrasse, Munich, Germany; 4 Ca de Massa, Llesp – El Pont de Suert, Spain; 5 Museu de Ciències Naturals, Barcelona, Spain

## Abstract

For management strategies in the context of global warming, accurate predictions of species response are mandatory. However, to date most predictions are based on niche (bioclimatic) models that usually overlook biotic interactions, behavioral adjustments or adaptive evolution, and assume that species can disperse freely without constraints. The deep subterranean environment minimises these uncertainties, as it is simple, homogeneous and with constant environmental conditions. It is thus an ideal model system to study the effect of global change in species with poor dispersal capabilities. We assess the potential fate of a lineage of troglobitic beetles under global change predictions using different approaches to estimate their thermal niche: bioclimatic models, rates of thermal niche change estimated from a molecular phylogeny, and data from physiological studies. Using bioclimatic models, at most 60% of the species were predicted to have suitable conditions in 2080. Considering the rates of thermal niche change did not improve this prediction. However, physiological data suggest that subterranean species have a broad thermal tolerance, allowing them to stand temperatures never experienced through their evolutionary history. These results stress the need of experimental approaches to assess the capability of poor dispersal species to cope with temperatures outside those they currently experience.

Climate change has become one of the main threats to global biodiversity^1^. For management strategies in the context of global warming, accurate predictions of species response are mandatory. However, to date most assessments of species’ vulnerability under climate change have been derived from niche (bioclimatic) models^3^. These approaches relate observed geographic distribution of a species to current climate; resultant models are then applied to climate projections to infer potential climatically suitable areas for a given species in the future. This allows detecting populations likely to remain stable over coming decades, as compared with others that are likely to be lost^4^,^5^,^6^. However, the application of these approaches has been debated as they present some important uncertainties^7^. It is assumed that i) species are able to disperse freely without any constraint; ii) are perfectly adapted to the environmental conditions of their current distributions; iii) the variables determining species distribution are known; iv) environmental conditions (usually estimated for grid cells at different spatial resolution) are homogeneous through the habitat, ignoring both temporal (daily and often seasonal) and spatial (micro-habitat) heterogeneity^9^,^10^,^11^,^12^; and v) organisms have no control over the conditions to which they are exposed, ignoring both behavioural and phenological accommodation at different environmental conditions^13^,^1^4 and the possibility of evolutionary adaptation^15^,^16^,^17^.

There is, however, a system in which all these uncertainties are minimised: the deep subterranean environment. Contrary to what happens in epigean (surface) environments, the range of variables affecting species in this environment is minimal. The humidity in the deep parts of a cave is always near the saturation point and the temperature is highly constant through the day and the year. It is also possible to have a reliable estimate of this temperature, which is approximately equal to the mean annual temperature of the surface^18^,^19^,^20^ (see Supplementary Fig. S1). Besides, environmental conditions are practically homogeneous through all possible microhabitats within a cave system, so small-scale spatial heterogeneity and the possibility of behavioural adjustments or phenotypic plasticity are greatly reduced. Caves harbour very simple biological communities^20^, minimising the need to account for biological interactions. In sum, unlike in surface environments, here we know the environmental conditions that species experience. This, together with the availability of almost complete phylogenies for some lineages, allows tracing their geographical movements through several cladogenetic processes and estimating rates of evolution. Therefore, deep subterranean species are an ideal model group for ecological, evolutionary and biogeographical studies^20^,^21^.

There is, however, one assumption that these species clearly violate, as they have a limited capacity for dispersal^20^. They cannot be expected to occupy much of their suitable habitat at short time scales due to their reduced mobility, so their only possibility to cope with fast climate change is to persist *in situ*. This makes them a particularly well suited group to evaluate the effect of predicted global change, as they cannot accommodate to changing conditions by behavioural plasticity, microhabitat use or by migrating to more suitable areas.

Here we aim to assess the capability to persist in place in a changing world of a lineage of 85 species of highly specialized troglobitic beetles of the tribe Leptodirini living in the North-eastern Iberian Peninsula. We used a range of methods to estimate their thermal niche breadth, as temperature is likely to be the most significant of the relatively few environmental variables affecting species in this environment. We first used a traditional approach based on current distributions and climate data, but including present, past and predicted future climatic conditions. Using comprehensive species level phylogenies, we also calculated the minimum range of temperatures they should have experienced through their evolutionary history, and the associated rates of minimum thermal niche change. We then consider the estimated thermal niche from experimentally determined thermal tolerances data. Finally, we compare these results with the predicted climatic conditions in 2080 for the same areas and discus the suitability of all these approaches to estimate thermal niche and the capability of poor dispersal species to cope with climate change.

## Results

### Current and Last Glacial Maximum (LGM) temperature ranges

**Figure 1:**
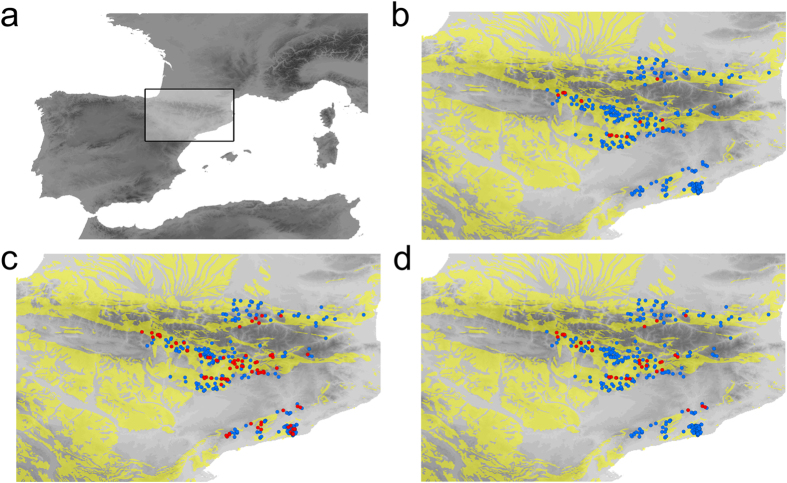
Study area. (**a**) Points mark the placement of caves with known populations of any of the species of the studied lineage. In yellow, areas with karstic substratum, i.e. suitable to have deep subterranean environment. In red, caves with predicted suitable current conditions when the thermal niche of the species was estimated from the LGM temperatures (**b**) and those predicted to have suitable conditions in 2080 under the B2 (**c**) and A2 (**d**) scenarios when the thermal niche was estimated from current temperatures. Maps were created using ArcGIS software by Esri (Environmental Systems Resource Institute, ArcMap 10.1, (www.esri.com).

We could georeference 403 caves for 83 of the 85 species of the clade (Fig. 1), with a maximum number of 104 caves for the same species (*Troglocharinus ferreri* (Reitter)), and up to 3 species known from the same cave. The temperatures of the caves occupied by the species of the studied clade ranged from ca. 1–3 °C in some species of *Trapezodirus* to just above 16 °C in *T. ferreri*, with an estimated divergence between these two taxa and their most recent common ancestor of ca. 10MY (see Supplementary Fig. S2 and Table S2). The average temperature range to which each of the studied cave species are currently exposed is ca. 3 °C, with a maximum value of 7.2 °C for *Trapezodirus bolivari* (Escalera) (from 2.40–9.60 °C; Supplementary Table S3). The estimated ranges for the reconstructed LGM temperatures of the same caves were very similar (maximum value, also with *T. bolivari*, (from −2.50–4.7 °C; Supplementary Table S3).

**Figure 2:**
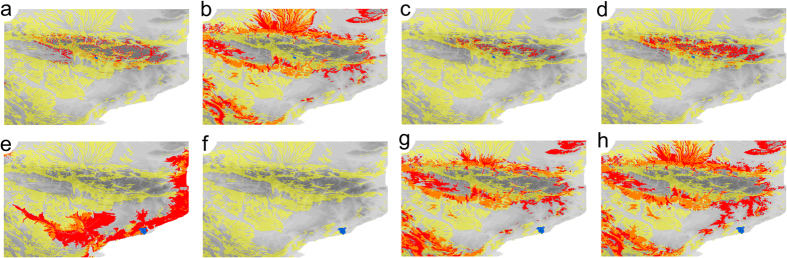
Thermal niche of some exemplar species estimated from distribution and climate. In yellow, areas with karstic substratum, i.e. suitable to have deep subterranean environment. In red, areas with suitable temperature for *Trapezodirus cerberus* (**a–d**) and *Troglocharinus ferreri* (**e–h**). The overlapping between the two (i.e. karstic areas with suitable temperatures) is represented in orange. Blue dots indicate known current distribution. (**a,e**) Current potential distribution estimated with current climatic conditions; (**b,f**) potential distribution during the LGM estimated from current climatic conditions; (**c,g**) current potential distribution estimated from the LGM climatic conditions; (**d,h**) potential distribution in 2080 (scenario A2) estimated with current climate. Maps were created using ArcGIS software by Esri (Environmental Systems Resource Institute, ArcMap 10.1, (www.esri.com).

For most species, bioclimatic models showed that suitable temperatures (defined as temperatures within the range they currently experience) during the LGM were estimated to occur in karst areas separated from their current ranges by non-suitable habitat patches, that is, by areas without deep subterranean environment. Only 7 species had a locality (37 localities in total) with an estimated LGM temperature within the range of their current temperatures. In some cases, suitable conditions were even non-existent in the whole northeast Iberian Peninsula, as for example the coastal species of *Troglocharinus* (Fig. 2). To track localities within their current range of temperatures these species would have had to disperse through areas considered to be unsuitable, without subterranean environment, in some cases well outside the current distribution of the whole clade. It can thus be safely concluded that to survive through the LGM (and likely at least several previous glacial periods) most species had to withstand temperatures outside the range they currently experience.

**Figure 3:**
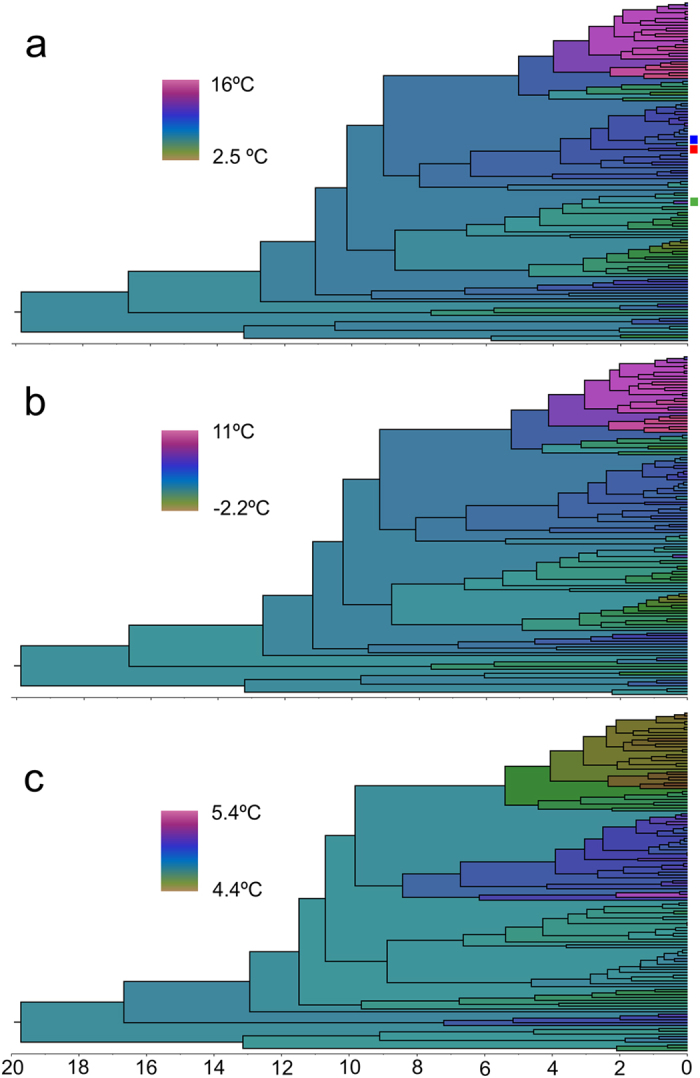
Phylogeny of the studied lineage obtained and reconstruction of the temperature change. Reconstructions were done using (**a**) the current temperature of the localities; (**b**) the estimated temperatures during the Last Glacial Maximum; and (**c**) the maximum historical range of temperature (Last Inter Glacial-Last Glacial Maximum) experienced in each of the caves. Horizontal axes, time before present (Ma). The position of *Trapezodirus orobios, Speonomus longicornis* and *S. fagniezi* Jeannel was indicated by green, red and blue squares, respectively. See Supplementary Fig. S2 for details of the phylogeny.

The reconstruction of the minimum temperature change in the phylogeny using current or estimated LGM temperatures were almost indistinguishable (Fig. 3), with a difference between 4.4–5.5 °C. This difference was not homogeneously distributed through the phylogeny, with the species under warmer current conditions having experienced a narrower historical range of temperature change (Supplementary Fig. S3). Only 19 localities of 7 different species were estimated to have been at average below zero temperatures during the LGM (Supplementary Table S2). All but three of these 7 species are known from additional close localities estimated to have been above zero during the LGM.

### Predicted temperature ranges

Under the A2 scenario of climate change (see Methods), in 2080 only 9% of the extant localities would remain at temperatures within the current range of their respective species; this percentage was raised to 26% under the B2 scenario (Fig. 1c,d; Supplementary Table S2). This translates into a maximum of either 26 or 48 species predicted to have at least one population with suitable temperatures for the A2 and B2 scenarios respectively, and only 8 or 16 species with more than 50% of their current populations predicted to have suitable temperatures in 2080.

### Thermal niche change

The mean rate of minimum absolute temperature change across all the branches of the tree was 0.76 °C/MY. The species that experienced the overall fastest minimum rate of absolute thermal niche change when considering only current conditions was *Trapezodirus orobios* (Fresneda, Hernando & Lagar), with an average of 0.84 °C/MY (Fig. 3a). The branch of the tree with the highest absolute rate of thermal niche change was found in *Speonomus longicornis* (Saulcy), with a rate of 3.62 °C/MY (from 9.42–8.2 °C in the last 0.33MY). The fastest rate of change when considering only a temperature increase was 2.28 °C/MY in a terminal branch of *Speonomus fagniezi* Jeannel (from 10.9–12.9 °C in 0.87MY). When we considered the estimated LGM paleotemperatures of the areas currently occupied by the species, the maximum rate of thermal niche change was found in a population of *Trapezodirus orobios*, from 4.4 °C to 10.4 °C in the 21,000 years since the estimation used for the LGM (i.e., 0.029 °C/100 years).

The IPCC estimated rates are 3–4 °C for mean annual temperature in the next ~100 years, depending on location (IPCC 2007). The minimum rate of change estimated among the species of the clade, even considering the constraint of Quaternary glaciations, was then between ~10,000 and ~100 times slower than the expected rate of change from 2000–2100.

### Thermal niche determined by physiological data

All current populations of the species for which there is available data^22^ are predicted to have temperatures within their physiological tolerances in 2080, both under the A2 and B2 scenarios. Only some coastal populations of *Troglocharinus* are expected to be close to their upper thermal limit (Supplementary Fig. S4 and Table S2).

## Discussion

When we predicted the fate of the studied subterranean species under a global change scenario using their current distribution to estimate their thermal niche, results were dramatic. Most areas with predicted suitable temperatures fall outside their current distribution, and given the low mobility of the species, dispersal to other areas seems unlikely. These results are comparable to those obtained in most of the studies using similar methodologies, which predict large geographic displacements and widespread extinctions^23^,^2^4. It has been argued that species could stand the predicted change in place through adjustments in microhabitat use, phenology or behaviour^12^,^2^5, but in our case, although minimal variation in humidity or airflow could affect in some degree the distribution of fauna within the cave, the possibility of these adjustments is very limited due to the general homogeneity of their environments. However, we show that the use of thermal niches estimated from LGM climatic conditions (Fig. 2c,g) failed to predict the response to past climate changes, as only 4% of the populations of the LGM were expected to maintain suitable conditions through the Holocene (Fig. 1b) - which obviously was not the case. From our results it can be confidently concluded that in this group species maintained their ranges through the last glacial cycle, given the impossibility to track optimal climatic conditions by dispersing to areas with suitable temperatures. It is thus clear that based only on current conditions it is not possible to predict the fate of this clade of subterranean species in front of predicted global change.

If species have difficulties in tracking their optimal temperatures and cannot exploit habitat heterogeneities, they may adapt to the new conditions through the evolution of their thermal tolerances^25^,^2^6. However, our results suggest that to survive *in situ* the projected changes in climatic conditions subterranean species would require rates of climatic niche evolution that are largely unprecedented in their evolutionary history. Similar results were found in a recent study^17^ using 17 clades of terrestrial vertebrates, although in this case only sister species were considered and thus the estimated changes were restricted to the most recent cladogenetic events. We applied a phylogenetic method to reconstruct temperature changes without any internal constraints other than the evolutionary model used, which means that we minimised the global amount of change through the evolutionary history of the lineage. It could be possible to envisage alternative scenarios with an unequal distribution of change, which could be concentrated on some branches thus increasing the maximum estimated rates. But this would also require that a high proportion of the species were able to closely track their optimal temperatures to minimise change even further, which seems highly unlikely given our results. In any case, differences in estimated past and future rates of change were of such magnitude that accounting for potential evolution did not improve the predicted demise of these species.

To reconstruct the temperatures during the LGM we used a global model^27^, with an estimated age of 21 ka BP. However, the growing consensus is that the glacial maximum (defined as the maximum extent of the ice cover) occurred earlier in the Iberian Peninsula than in the rest of Europe, at ca. 50 ka BP^28^. This should not affect our conclusions, as the estimated absolute range of temperature differences remains the same and the increase in the transition time between glacial and interglacial can only lower the rates of change.

As a direct consequence of the stability of the subterranean environment, the reconstructed minimum change of thermal tolerance experienced by the studied lineage through their evolutionary history (approximately ±5 °C) was surprisingly low. Even so, this may have been enough to cope with climate change in the last ca. 18 MY. If species maintained a window of thermal tolerance, or a range of phenotypic plasticity, wider than these ±5 °C, a possibility is that they did not experience the need to adjust to climatic changes. Thermal tolerance experiments with some of the species of the studied lineage suggest that this may be the case^22^. The upper limit of this range (20 °C) would be above the maximum temperatures that any of the species of the clade may have experienced since the Miocene. This lack of adjustment to local temperature was interpreted as a result of the loss of costly regulatory mechanisms due to the extreme conditions of the deep subterranean environment^22^.

Our results suggest that for species with poor dispersal capabilities, and even under optimal circumstances minimising the uncertainties of bioclimatic models, only an experimental approach combined with data on species biology^14^,^2^9 can provide strong evidence of the capability of species to cope with temperatures outside those of their current environment. In the case of the studied lineage of exclusive deep subterranean species, we can say that most species will be exposed to climatic conditions and rates of change unprecedented in their evolutionary history, but that in most cases temperatures will still be within their fundamental niche due to the lack of adjustment to local conditions. However, this lack of adjustment also means that for those species already close to the upper limit of their fundamental niches (i.e. those with the lower Thermal Safety Margins; TSM) the possibilities of survival are severely limited. To what extent this is a general situation or just a peculiarity of subterranean species is not known, as physiological data is still available for only a very limited number of species and the possibility of behavioural or phenological adjustments is usually not considered.

Species living in the warmer areas close to the coast seem to be most vulnerable, as temperature for these areas is predicted to be around 20 °C in 2080 (see Supplementary Fig. S4). This is close to the estimated upper limit of their fundamental climatic niche^22^ thus limiting their thermal safety margin. In general, conservation recommendations related to climate change are mostly strategies based on the selection of protected areas and measures to increase habitat connectivity^30^,^31^. However, for cave species, as happens for other species with poor dispersal capabilities, the concentration of conservation efforts in actual localities (i.e., *in situ* management) could be a more efficient and practical strategy. Although these measures are recommended for species living in the central Pyrenees (i.e. those with wider TSM), they seem insufficient for species and populations highly vulnerable to climatic change, such as those living in the coast. At present it is not know whether the situation of the coastal species of *Troglocharinus* is common among the subterranean fauna or a unique situation consequence of their peculiar evolutionary history^32^. Despite the long fascination by the peculiarities of typical subterranean organisms^33^,^34^,^35^ there is a general lack of knowledge of most subterranean groups worldwide. Subterranean biodiversity has been largely neglected in conservation programs worldwide, even if it has been estimated a total of 50,000–100,000 obligate subterranean species^36^, with a high level of local endemism^37^.

Our results suggest that assessments of global warming impacts for poor dispersal species based only on bioclimatic models could result in significant prediction errors, perhaps underestimating the persistence of species *in situ* and overestimating their potential to access and exploit predicted future climate space^7^,8,^3^8. These same approaches have been applied to many species with narrow distribution for which dispersal ability or thermal tolerance are not known resulting in some cases in alarming predictions. To deal with these uncertainties, a new, integrated science of climate-change biodiversity assessment is emerging based on multiple sources and approaches^39^.

## Materials and Methods

We focused on a well-defined clade of troglobitic beetles of the tribe Leptodirini (Coleoptera, Leiodidae) living in the North-eastern Iberian Peninsula (from the Pyrenees to the coast of Catalonia; Fig. 1a), including 85 species (Supplementary Table S1). Note that we defined our “species” as monophyletic units, which in some cases include lineages within non monophyletic species, subspecies or more than one species when one of them occurs in a single cave to increase the sample size for modelling purposes (see Supplementary Table S1). The studied clade was reconstructed to have a highly modified life cycle^40^ and all species have the typical troglobitic morphological characters (i.e. they are blind, apterous, depigmented and with elongated appendages)^41^. Most of them are narrow endemic species with a well-known distribution^41^.

### Estimating thermal niche (and potential distribution) using distribution and climatic data

For each of the taxa we compiled georeferenced distributional data from our own records, specialized publications and other unpublished information to estimate the extremes of the climatic conditions within their entire ranges. To characterise current thermal niches we used the mean annual temperature from the entire study area at 0.08 degree spatial resolution cells from WORLDCLIM version 1.3 (http://www.worldclim.org)^42^.

To compute the bioclimatic models, we first estimated for each species the temperature in the observed presence localities (i.e. the estimated temperature of the cave), and then calculated the extreme climatic values for their whole range. Then, assuming that temperature is the most important factor to characterise their climatic niche and that these recorded occurrences are representative of the full climatic conditions in which the species may survive and reproduce, we used these extreme values to derive a distributional hypothesis on the areas with climatically (thermal) suitable conditions (considered here as the potential distribution)^7^. Basically, all grid cells with climatic values falling within the mentioned range were designated as suitable, and all cells outside it as unsuitable. We use this “bioclimatic envelope” procedure to be conservative, as it is an established procedure directed at maximizing the capacity to represent geographically the potential distribution of species niches when they are only based on distributional data^43^.

### Estimation of the range of temperatures to which species were exposed in the past

We followed the same approach to estimate the thermal niche and potential distribution of the species using paleoclimatic data for the Last Glacial Maximum (LGM; 21,000 YBP). For the estimation of the LGM temperatures we used a simulation of the general circulation model (GCM) from the Community Climate System Model (CCSM, www.ccsm.ucar.edu)^27^. The original GCM data were downloaded from the PMIP2 website (http://www.pmip2.cnrs-gif.fr/).

### Thermal niche changes

#### Taxon sampling, sequencing

We used molecular data from previous studies^40^,^44^,^45^, completed with 51 new specimens of 27 additional taxa as well as specimens from different caves for the species with the widest distributions. The final matrix for the phylogenetic analyses included a total of 122 specimens of 64 species in 18 genera (out of the 85 known^40^,^41^; see Supplementary Table S2).

Specimens were collected by direct search or with the use of baits and stored in absolute ethanol. DNA extractions of single specimens were non-destructive, using either a phenol–chloroform method or commercial kits (mostly DNeasy Tissue Kit, Quiagen, Hilden, Germany) following the manufacturer’s instructions. Vouchers are kept at the Museo Nacional de Ciencias Naturales, Madrid (MNCN) and the Institute of Evolutionary Biology, Barcelona (IBE). We amplified and sequenced the same fragments as in a previous study^40^, i.e. seven genes totalling more than 4 Kb: five mitochondrial (cox1 –amplified in two fragments–, cob, rrnL, trnL, nad1 –the last three amplified in a continuous single fragment) and two nuclear (SSU, LSU). Primers and PCR conditions were the same as in a previous study^44^. New sequences (129) have been deposited in GenBank (EMBL) with Acc. Nos. LN849257-LN849385 (Supplementary Table S2).

#### Phylogenetic analyses

We aligned length variable (i.e. ribosomal) sequences with MAFFT v. 7 and the Q-INS-i algorithm, which considers the secondary structure^45^. To obtain an ultrametric tree we used BEAST v.1.7^46^ with an uncorrelated lognormal clock and a Yule speciation process. We linked the evolutionary rates of the mitochondrial protein coding genes and the two nuclear ribosomal genes respectively to reduce the number of free parameters, and used a GTR + I + G model with unlinked parameters for each partition. Trees were rooted in the node separating a clade with 1 instar larval development from a clade with either 2- or 1-instar, according to the topology of a previous study^39^ (Supplementary Fig. S2). For the calibration we used the estimates obtained in this same study, that were based on the tectonic separation of the Sardinian plate, 16.6 Ma for the clade of species with a 1-instar cycle and 13.7 Ma for the clade of uncertain life cycle type. Both were set up as priors with a normal distribution with the estimated age as mean and a standard deviation of 1 MY.

For the estimation of the minimum accumulated change in temperature experienced through the evolutionary history of the lineage, we used ancestral trait reconstruction in BEAST, using as a quantitative trait the temperature of the cave in which each specimen was found (either directly measured or inferred from the annual average temperature of the locality; see Supplementary Tables S2 and S3), and a multivariate diffusion model of evolution. We used three different measures: current temperature, the estimated LGM temperature, and the difference between the two (“range”) (see below). Analyses were run for 60 million generations, with a conservative burn-in of 10 millions. In all Bayesian analyses convergence was assessed with the effective sample size in TRACER 1.5. This reconstruction can be interpreted as the minimum amount of change in the thermal niche that the species should have experienced through their evolutionary history given the observed differences between species and the reconstructed topology.

#### Estimating rates of thermal niche change

To estimate the minimum rates of thermal niche change that the species must have experienced we used three measures: i) average rate of thermal niche change, calculated as the average rate of all branches connecting all the specimens of each species with the root of the tree. For each branch, rate evolution was calculated as the absolute difference between the reconstructed initial and final temperatures divided by the length of the branch. ii) Maximum per-branch rate of thermal niche change, or the highest per-branch reconstructed rate among all the branches of the tree. iii) Adjusted LGM rate of change, calculated as the difference between the estimated paleotemperatures of the areas occupied by the species during the LGM (21,000 YBP) and the current temperatures. This range was estimated according to our conclusion that the species experienced this variation *in situ* (see Results above) (Figs 1b and 2b,f).

We also estimated how many of the current localities in which each species occur would have suitable thermal conditions in 2080 (see Fig. 2d,h), following the same procedure as before but projecting the thermal niche of each species to the predicted temperature for 2080 according to the B2 and A2 scenarios for the CCCMA-CGCM2 climate model through the CIAT database (www.ccafs-climate.org), and at the same spatial resolution.

### Thermal tolerances determined by physiological experiments

We obtained thermal limits from a recent study^22^. Among others, they conducted experiments of survival at different temperatures for up to 7 days for three of the species of this same clade. They used species under different thermal conditions since the Pliocene occupying the environmental range of the clade. They showed that both the Pyrenean and coastal species of the lineage seemed to be able to stand temperatures between approximately 1–20 °C, but in all cases they did not survive more than 48 h at 23 °C^22^. In short term experiments, the lower thermal limit (supercooling) was found to be ca. −2.5 °C, close to the estimated freezing temperature of hemolymph in the absence of cryoprotectants.

## Additional Information

**How to cite this article**: Sánchez-Fernández, D. *et al.* Thermal niche estimators and the capability of poor dispersal species to cope with climate change. *Sci. Rep.* **6**, 23381; doi: 10.1038/srep23381 (2016).

## Supplementary Material

Supplementary Information
