# Supplementary material for: Thermal niche estimators and the capability of poor dispersal species to cope with climate change
Source: Sci Rep. 2016 Mar 17;6:23381. doi: 10.1038/srep23381 (PMC4794760; doi:10.1038/srep23381)
Supplement: Supplementary Information [file srep23381-s1.doc]

**SuppLEMENTARY Information**

**Thermal niche estimators and the capability of poor dispersal species to cope with climate change**

David Sánchez-Fernández, Valeria Rizzo, Alexandra Cieslak, Arnaud Faille, Javier Fresneda and Ignacio Ribera

**Figure S1 Relationship between the temperature of the cave and the surface.** The temperature of the cave can be estimated with an average error of 1.90 °C using as predictor only a raster with the Mean Annual Temperature of the surface (R² = 0.61, n=27; p <0.01). Regression line is represented in red and equality line in green.


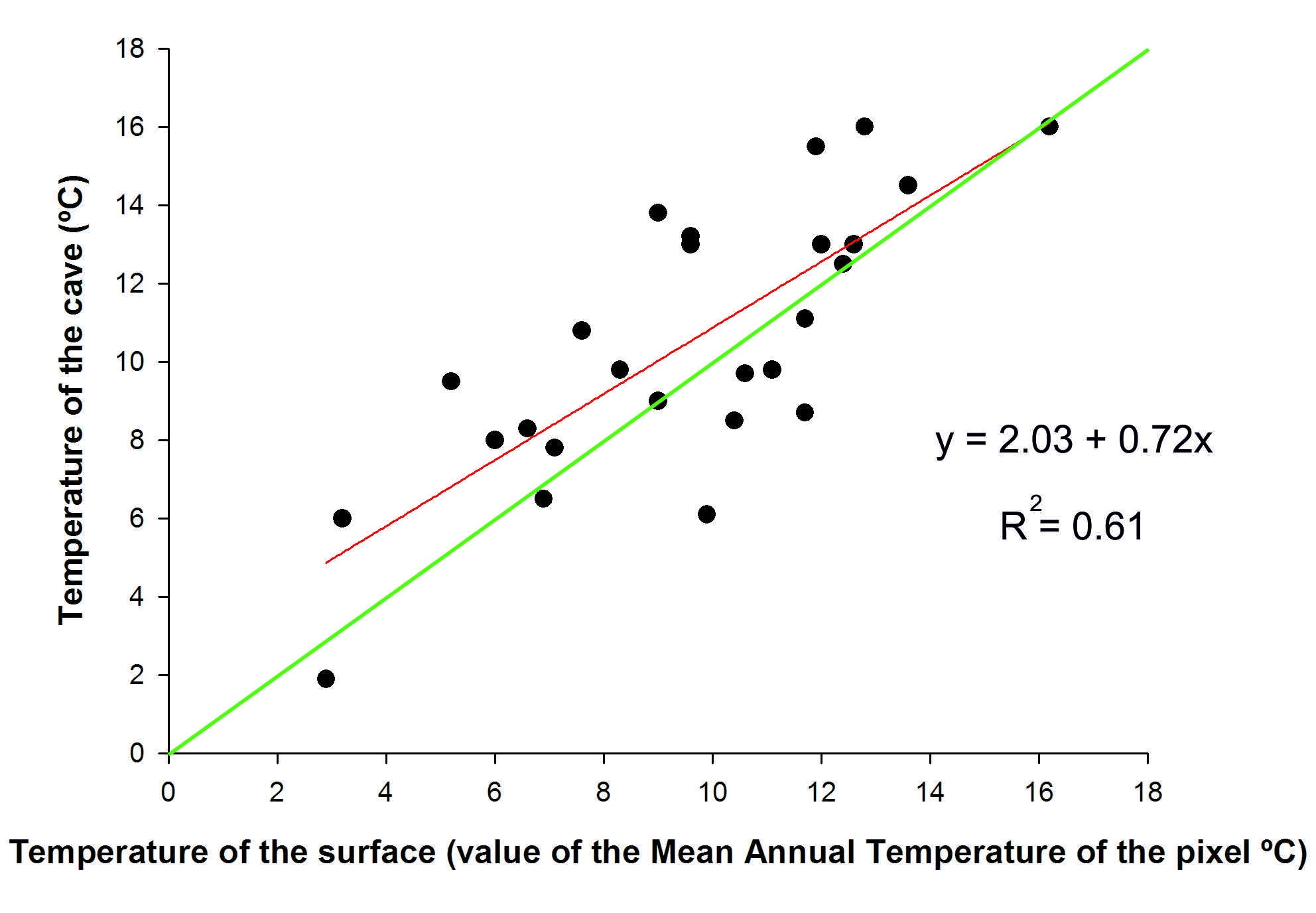


**Figure S2 Phylogeny of the studied lineage obtained with BEAST.** Numbers in nodes, posterior probabilities. With black circles, nodes used in the calibration of the phylogeny, with the used ages (from Cieslak *et al*. 2014). Horizontal axis, time before present (Ma). See Supplementary Table S2 for details of the specimens.

**

**

**Figure S3** **Relationship between the difference of Mean Annual Temperatures (MAT) from LGM to current conditions (MAT (current-LGM)) and the mean annual temperature (MAT (current)) estimated for each species.** The species under warmer current conditions have experienced a narrower historical range of temperature change (r^2^= 0.14; p<0.01).


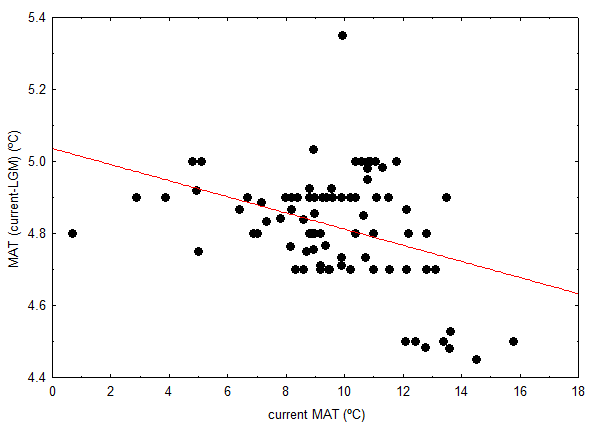


**Figure S4 Past, current and future temperature in the study area.** Mean Annual Temperature in the study area estimated for a) the Last Glacial Maximum, b) present and future scenarios c) B2 and d) A2 (see main text for references). Points mark the placement of caves with known populations of any of the species of the studied linage. Maps were created using ArcGIS software by Esri (Environmental Systems Resource Institute, ArcMap 10.1, (www.esri.com).

**
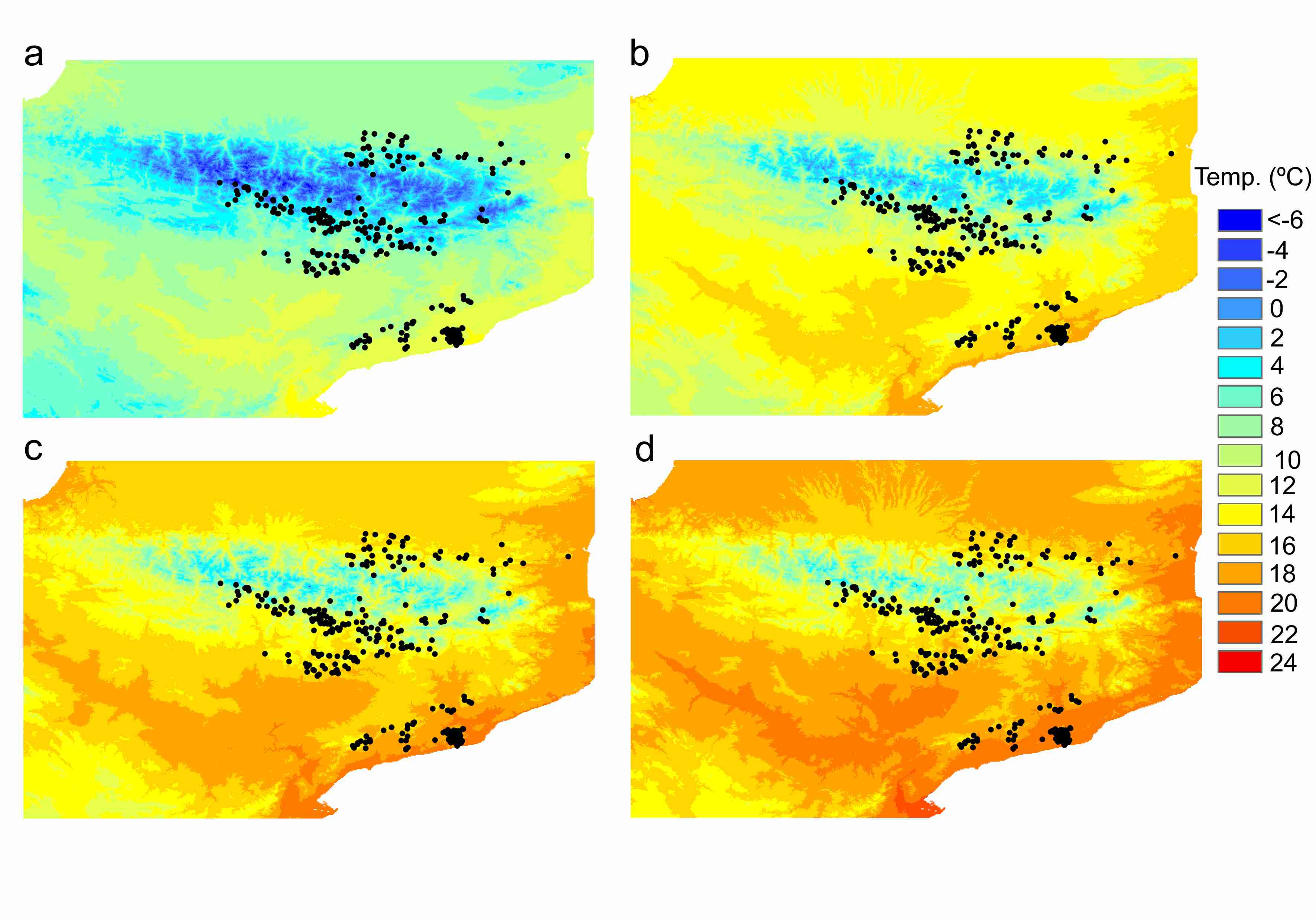
**

**Table S1** **Taxonomic composition of the studied clade.** Taxon, species and subspecies as currently recognized (see Salgado et al. 2008). Taxonomic species, species as currently recognized (i.e. without subspecies). Coded_species, monophyletic units used (i.e. "species" through the text). As noted in the main text, different lineages of non-monophyletic species or subspecies, plus in some cases species with a single geo-referenced cave, may be grouped into the same monophyletic unit or “Coded_species”. With asterisks, species for which data of geo-referenced caves could not be obtained.

| Taxon | Taxonomic  species | Phylogenetic  species | Coded_species | Taxon name |
| --- | --- | --- | --- | --- |
| 1 |  | 1 | Ant_dispar | *Antrocharis querilhaci dispar* Abeille de Perrin, 1878 |
| 2 | 1 | 2 | Ant_querilhaci | *Antrocharis querilhaci querilhaci* (Lespès, 1857) |
| 3 | 2 | 3 | Cer_cenarroi | *Ceretophyes cenarroi* (Español, 1955) |
| 4 | 3 | 4 | Cer_riberai | *Ceretophyes riberai* (Español, 1967) |
| 5 | 4 | 5 | Ges_delioti | *Gesciella delioti* Giachino & Gueorguiev, 1989 |
| 6 | 5 | 6 | Lag_colominasi | *Lagariella colominasi* (Zariquiey, 1924) |
| 7 | 6 | 7 | Lag_porroinensis | *Lagariella porroiensis* (Escolà & Comas, 1983) |
| 8 | 7 | 8 | Met_monticola | *Metaspeonomus monticola* Coiffait 1959 |
| 9 | 8 | 9 | Nas_eseranus | *Naspunius eseranus* (Lagar, 1974) |
| 10 | 9 | 10 | Pal_pallaresana | *Pallaresiella pallaresana* (Jeannel, 1911) |
| 11 | 10 | 11 | Par_carrerei | *Paratroglophyes carrerei* Fourès, 1954 |
| 12 | 11 | 12 | Par_jeanneli | *Paratroglophyes jeanneli* Coiffait, 1955 |
| 13 | 12 | 13 | Par_orestes | *Paratroglophyes orestes* Fresneda, Bourdeau & Faille, 2011 |
| 14 | 13 | 14 | Pas_vandeli | *Paraspeonomus vandeli* Coiffait 1952 |
| 15 | 14 | 15 | Per_bofilli | *Perriniella bofilli* Zariquiey, 1924 |
| 16 | 15 | 16 | Per_faurai | *Perriniella faurai* Jeannel, 1910 |
| 17 | 16 | 17 | Per_fresnedai | *Perriniella fresnedai* Perreau & Tronquet, 2001 |
| 18 | 17 | 18 | Sal_brieti | *Salgadoia brieti* (Jeannel, 1911) |
| 19 | 18 | 19 | Spe_abeillei | *Speonomus (Speonomus) abeillei abeillei* (Saulcy, 1872) |
| 20 |  |  | Spe_abeillei | *Speonomus (Speonomus) abeillei bouilloni* Coiffait, 1959 |
| 21 | 19 | 20 | Spe_bonvouloiri | *Speonomus (Speonomus) bonvouloiri* (Jacquelin du Val, 1859) |
| 22 | 20 | 21 | Spe_carrerei | *Speonomus (Speonomus) carrerei* Fourès, 1954 |
| 23 |  | 22 | Spe_chardonis | *Speonomus (Speonomus) chardonis aletinus* (Abeille de Perrin, 1883) |
| 24 | 21 |  | Spe_chardonis | *Speonomus (Speonomus) chardonis chardonis* (Abeille de Perrin, 1875) |
| 25 |  |  | Spe_chardonis | *Speonomus (Speonomus) chardonis hecatae* (Abeille de Perrin, 1878) |
| 26 |  |  | Spe_chardonis | *Speonomus (Speonomus) chardonis pueli* (Chobaut, 1903) |
| 27 | 22 | 23 | Spe_colluvii | *Speonomus (Speonomus) colluvii* Delay, Juberthie & Ruffat, 1983 |
| 28 | 23 | 24 | Spe_curvipes | *Speonomus (Speonomus) curvipes curvipes* (Piochard-de-la-Brûlerie, 1872) |
| 29 |  |  | Spe_curvipes | *Speonomus (Speonomus) curvipes subcurvipes* (Abeille de Perrin, 1878) |
| 30 |  |  | Spe_curvipes | *Speonomus (Speonomus) curvipes subrectipes* (Abeille de Perrin, 1878) |
| 31 | 24 | 25 | Spe_diecki | *Speonomus (Speonomus) diecki* (Saulcy, 1872) |
| 32 | 25 | 26 | Spe_fagniezi | *Speonomus (Speonomus) fagniezi* Jeannel, 1910 |
| 33 |  | 27 | Spe_longicornis | *Speonomus (Speonomus) longicornis fauveaui* (Jeannel, 1907) |
| 34 |  |  | Spe_longicornis | *Speonomus (Speonomus) longicornis fuxeensis* Jeannel, 1908 |
| 35 |  |  | Spe_longicornis | *Speonomus (Speonomus) longicornis hermensis* (Abeille de Perrin, 1873) |
| 36 | 26 |  | Spe_longicornis | *Speonomus (Speonomus) longicornis longicornis* (Saulcy, 1872) |
| 37 |  |  | Spe_longicornis | *Speonomus (Speonomus) longicornis pandellei* (Abeille de Perrin, 1883) |
| 38 |  |  | Spe_longicornis | *Speonomus (Speonomus) longicornis perieri* (Piochard-de-la-Brûlerie, 1872) |
| 39 |  | 28 | Spe_normandi | *Speonomus (Speonomus) normandi bergesi* Coiffait, 1959 |
| 40 |  |  | Spe_normandi | *Speonomus (Speonomus) normandi hydrophilus* (Jeannel, 1907) |
| 41 | 27 |  | Spe_normandi | *Speonomus (Speonomus) normandi normandi* (Jeannel, 1906) |
| 42 | 28 | 29 | Spe_piochardi | *Speonomus (Speonomus) piochardi* (Abeille de Perrin, 1873) |
| 43 | 29 | 30 | Spe_proserpinae | *Speonomus (Speonomus) proserpinae* (Abeille de Perrin, 1878) |
| 44 |  | 31 | Spe_pyreneus | *Speonomus (Speonomus) pyreneus discontignyi* (Saulcy, 1872) |
| 45 |  |  | Spe_pyreneus | *Speonomus (Speonomus) pyreneus hustachei* Coiffait, 1959 |
| 46 |  |  | Spe_pyreneus | *Speonomus (Speonomus) pyreneus major* Coiffait, 1959 |
| 47 |  |  | Spe_pyreneus | *Speonomus (Speonomus) pyreneus maurasi* Coiffait, 1955 |
| 48 |  |  | Spe_pyreneus | *Speonomus (Speonomus) pyreneus nadari* (Jeannel, 1906) |
| 49 |  |  | Spe_pyreneus | *Speonomus (Speonomus) pyreneus nerzici* Coiffait, 1959 |
| 50 |  |  | Spe_pyreneus | *Speonomus (Speonomus) pyreneus novemfontium* (Piochard-de-la-Brûlerie, 1872) |
| 51 |  |  | Spe_pyreneus | *Speonomus (Speonomus) pyreneus pratensis* Coiffait, 1959 |
| 52 | 30 |  | Spe_pyreneus | *Speonomus (Speonomus) pyreneus pyreneus* (Lespès, 1857) |
| 53 |  | 32 | Spe_stygius | *Speonomus (Speonomus) stygius brevicornis* Coiffait, 1952 |
| 54 |  |  | Spe_stygius | *Speonomus (Speonomus) stygius crassicornis* (Piochard-de-la-Brûlerie, 1872) |
| 55 |  |  | Spe_stygius | *Speonomus (Speonomus) stygius saulcyi* (Abeille de Perrin, 1872) |
| 56 | 31 |  | Spe_stygius | *Speonomus (Speonomus) stygius stygius* (Dieck, 1869) |
| 57 |  |  | Spe_stygius | *Speonomus (Speonomus) stygius thibali* Coiffait, 1959 |
| 58 |  |  | Spe_stygius | *Speonomus (Speonomus) stygius tisiphone* Jeannel, 1908 |
| 59 | 32 | 33 | Spe_zophosinus | *Speonomus (Speonomus) zophosinus* (Saulcy, 1872) |
| 60 | 33 | 34 | Spn_andorranus | *Speonomites andorranus* (Comas, 1978) |
| 61 | 34 | 35 | Spn_antemi | *Speonomites antemi* (Escolà, 1972) |
| 62 | 35 | 36 | Spn_aurouxi | *Speonomites aurouxi* (Español, 1966) |
| 63 | 36 | 37 | Spn_crypticola | *Speonomites crypticola* (Jeannel, 1910) |
| 64 | 37 | 38 | Spn_kryophilos | *Speonomites kryophilos* (Fresneda & Hernando, 1991) |
| 65 | 38 | 39 | Spn_latrunculus | *Speonomites latrunculus* (Jeannel, 1910) |
| 66 | 39 | 40 | Spn_leleupi* | *Speonomus (Speonomus) leleupi* Coiffait, 1953 |
| 67 | 40 | 41 | Spn_mengeli | *Speonomites mengeli* (Jeannel, 1910) |
| 68 | 41 | 42 | Spn_nitens | *Speonomites nitens* Jeannel, 1910 |
| 69 | 42 | 43 | Spn_tincatincensis | *Speonomites tincatincensis* (Escolà, Bellés & Comas, 1985) |
| 70 | 43 | 44 | Spn_torresi | *Speonomites torresi* (Fresneda & Hernando, 1990) |
| 71 | 44 | 45 | Spn_velox | *Speonomites velox* Jeannel, 1910 |
| 72 | 45 | 46 | Sty_akarsticus | *Stygiophyes akarsticus* (Escolà, 1980) |
| 73 | 46 | 47 | Sty_aldomai | *Stygiophyes aldomai aldomai* (Fresneda & Hernando, 1988) |
| 74 |  |  | Sty_aldomai | *Stygiophyes aldomai allomorphus* (Fresneda & Escolà, 2001) |
| 75 | 47 | 48 | Sty_espinosai | *Stygiophyes espinosai* (Bellés, 1983) |
| 76 | 48 | 49 | Sty_hansferyi | *Stygiophyes hansferyi* Fresneda & Escolà, 2001 |
| 77 |  | 50 | Sty_latebricola | *Stygiophyes latebricola elongatus* (Jeannel, 1911) |
| 78 | 49 |  | Sty_latebricola | *Stygiophyes latebricola latebricola* (Jeannel, 1911) |
| 79 | 50 | 51 | Sty_puncticollis | *Stygiophyes puncticollis* (Jeannel, 1910) |
| 80 | 51 | 52 | Sty_ribagorzanus | *Stygiophyes ribagorzanus* (Jeannel, 1911) |
| 81 | 52 | 53 | Sty_saforensis | *Stygiophyes saforensis* (Escolà, Bellés & Comas, 1985) |
| 82 | 53 | 54 | Sty_sanctigervasi | *Stygiophyes sanctigervasi* (Jeannel, 1911) |
| 83 | 54 | 55 | Sty_zariquieyi | *Stygiophyes zariquieyi* (Jeannel, 1924) |
| 84 | 55 | 56 | Tra_articollis | *Trapezodirus arcticollis* (Jeannel, 1911) |
| 85 | 56 | 57 | Tra_bolivari | *Trapezodirus bolivari* (Martínez de la Escalera, 1898) |
| 86 | 57 | 58 | Tra_carrodillae | *Trapezodirus carrodillae* (Jeannel, 1911) |
| 87 | 58 | 59 | Tra_cerberus | *Trapezodirus cerberus* (Jeannel, 1911) |
| 88 | 59 | 60 | Tra_escollae | *Trapezodirus escollae* (Fresneda & Hernando, 1994) |
| 89 | 60 | 61 | Tra_gimenezi | *Trapezodirus gimenezi* (Fresneda, Hernando & Lagar, 1998) |
| 90 | 61 |  | Tra_orobios | *Trapezodirus altimontanus* (Bellés, 1975) |
| 91 | 62 | 62 | Tra_orobios | *Trapezodirus orobios orobios* (Fresneda, Hernando & Lagar, 1998) |
| 92 |  |  | Tra_orobios | *Trapezodirus orobios robustus* (Fresneda, Hernando & Lagar, 1998) |
| 93 | 63 | 63 | Trc_mestrei | *Trocharanis mestrei* (Abeille de Perrin, 1878) |
| 94 |  | 64 | Tro_elongatus | *Troglocharinus elongatus abenzai* (Lagar, 1972) |
| 95 | 64 |  | Tro_elongatus | *Troglocharinus elongatus elongatus* Zariquiey, 1950 |
| 96 |  |  | Tro_elongatus | *Troglocharinus elongatus mateui* Zariquiey, 1950 |
| 97 |  |  | Tro_elongatus | *Troglocharinus elongatus ollai* Zariquiey, 1950 |
| 98 |  |  | Tro_elongatus | *Troglocharinus elongatus pinyareti* Zariquiey, 1950 |
| 99 |  |  | Tro_elongatus | *Troglocharinus elongatus portai* Zariquiey, 1950 |
| 100 | 65 | 65 | Tro_espanoli | *Troglocharinus espanoli* (Jeannel, 1930) |
| 101 |  | 66 | Tro_ferreri | *Troglocharinus ferreri abadi* Lagar, 1981 |
| 102 | 66 |  | Tro_ferreri | *Troglocharinus ferreri ferreri* (Reitter, 1908) |
| 103 |  |  | Tro_ferreri | *Troglocharinus ferreri pallaresi* Bellés, 1973 |
| 104 | 67 | 67 | Tro_fonti | *Troglocharinus fonti fonti* (Jeannel, 1910) |
| 105 |  |  | Tro_fonti | *Troglocharinus fonti infernus* (Jeannel, 1911) |
| 106 |  |  | Tro_fonti | *Troglocharinus fonti schuttei* (Español, 1955) |
| 107 |  |  | Tro_fonti | *Troglocharinus fonti zariquieyi* (Jeannel, 1924) |
| 108 | 68 |  | Tro_fonti | *Troglocharinus impellitieri* (Español, 1955) |
| 109 | 69 | 68 | Tro_hustachei | *Troglocharinus hustachei* Jeannel, 1911 |
| 110 | 70 | 69 | Tro_jacasi | *Troglocharinus jacasi* (Lagar, 1966) |
| 111 |  | 70 | Tro_kiesenwetteri | *Troglocharinus kiesenwetteri andresi* (Escolà, 1966) |
| 112 | 71 |  | Tro_kiesenwetteri | *Troglocharinus kiesenwetteri kiesenwetteri* (Dieck, 1869) |
| 113 |  |  | Tro_kiesenwetteri | *Troglocharinus kiesenwetteri sanllorensi* (Zariquiey, 1924) |
| 114 | 72 |  | Tro_kiesenwetteri | *Troglocharinus patracoi* (Zariquiey, 1922) |
| 115 | 73 | 71 | Tro_ludovici | *Troglocharinus ludovici* Bellés & Déliot, 1983 |
| 116 | 74 | 72 | Tro_olerdolai | *Troglocharinus olerdolai* Lagar, 1952 |
| 117 |  | 73 | Tro_orcinus | *Troglocharinus orcinus acevedoi* (Español 1953) |
| 118 |  |  | Tro_orcinus | *Troglocharinus orcinus lagari* (Español 1953) |
| 119 | 75 |  | Tro_orcinus | *Troglocharinus orcinus orcinus* (Jeannel 1910) |
| 120 | 76 | 74 | Tro_quadricollis | *Troglocharinus quadricollis* (Jeannel, 1911) |
| 121 | 77 | 75 | Tro_rovirai | *Troglocharinus rovirai* Lagar, 1975 |
| 122 | 78 | 76 | Tro_schibii | *Troglocharinus schibii* (Español, 1972) |
| 123 | 79 | 77 | Tro_senenti | *Troglocharinus senenti* Escolà, 1967 |
| 124 | 80 | 78 | Tro_subilsi | *Troglocharinus subilsi* (Español, 1966) |
| 125 | 81 | 79 | Tro_vinyasi | *Troglocharinus vinyasi* (Escolà, 1971) |
| 126 | 82 | 80 | Trp_aubryi | *Troglophyes aubryi aubryi* Coiffait 1953 |
| 127 |  |  | Trp_aubryi | *Troglophyes aubryi* vallierensis Coiffait, 1953 |
| 128 | 83 | 81 | Trp_bedeli | *Troglophyes bedeli* Jeannel 1906 |
| 129 |  | 82 | Trp_gavoyi | *Troglophyes gavoyi alluaudi* Jeannel 1911 |
| 130 | 84 |  | Trp_gavoyi | *Troglophyes gavoyi gavoyi* Abeille de Perrin, 1894 |
| 131 | 85 | 83 | Trp_ludovici | *Troglophyes ludovici* Chobaut 1903 |
| 132 | 86 | 84 | Trp_nsspecie | *Troglophyes* n. sp. |
| 133 | 87 | 85 | Trp_oblongus* | *Troglophyes oblongulus* Reitter, 1908 |

**Table S2** **Records used for this study with sequenced specimens and Accession Numbers**. Id_record, known species occurrences; Coded_species, code of the monophyletic units used (see Supplementary Table S1); Cave, Locality and Country-Province, name and and location of the cave; X, Y and Altitude, geographical coordinates (in decimal degrees) and altitude of the caves. Climatic conditions of the cave: MAT, Mean Annual Temperature of current, Last Glacial Maximum (LGM), Last Inter Glacial (LIG) conditions and predicted scenarios in 2080 (a2 and b2). Red columns, material used for the molecular analyses, including voucher reference, year, collector and accession numbers of the sequenced genes. Grey files represent repeated exemplars sequenced. In bold, newly obtained sequences.

| No | Id_record | Code_species | Id_cave | Cave | Locality | Country-Province | X | Y | Altitude | MAT (current) | MAT (LGM) | MAT (LIG) | MAT (b2) | MAT (a2) | voucher | year | leg | cox1 | 16S+tRNA-Leu+nad1 | cyb | 18S | 28S |
| --- | --- | --- | --- | --- | --- | --- | --- | --- | --- | --- | --- | --- | --- | --- | --- | --- | --- | --- | --- | --- | --- | --- |
| 1 | 1 | Trp_bedeli | 246 | Aven du Pla de Perillós | Opul | France-Pyrénées-Orientales | 2.86747 | 42.89486 |  | 13.5 | 8.6 | 13.6 | 15.9 | 17.1 |  |  |  |  |  |  |  |  |
| 2 | 2 | Spe_fagniezi | 506 | Grotte de l'Oratoire | Saint-Paul-de-Fenouillet | France-Pyrénées-Orientales | 2.48133 | 42.83758 |  | 12.9 | 8.1 | 13.1 | 15.4 | 16.6 |  |  |  |  |  |  |  |  |
| 3 | 3 | Spe_fagniezi | 505 | Grotte de la Madeleine | Saint-Paul-de-Fenouillet | France-Pyrénées-Orientales | 2.48123 | 42.83788 |  | 12.9 | 8.1 | 13.1 | 15.4 | 16.6 | IBE-AF125 | 2009 | C. Bourdeau | HF912456 | HF912608/HF912545 | HF912506 | HF912579 | HF912591 |
| 4 | 4 | Trp_gavoyi | 267 | Réseau Bufo Fret | Bugarach | France-Aude | 2.36931 | 42.86310 |  | 10.4 | 5.5 | 10.5 | 12.9 | 14.1 |  |  |  |  |  |  |  |  |
| 5 | 5 | Spe_bonvouloiri | 23 | Grotte de Fuilla | Villeneuve de Conflent | France-Pyrénées-Orientales | 2.35642 | 42.57864 | 467 | 12.8 | 8 | 12.9 | 15.2 | 16.4 | IBE-AC111 | 2009 | C. Vanderbergh | **LN849259** | - | **LN849301** | **LN849333** | **LN849343** |
| 6 | 6 | Trp_gavoyi | 247 | Aven du Col Saint Louis | Corbières sud, Saint Paul Fenouillet | France-Pyrénées-Orientales | 2.33092 | 42.83250 |  | 10.9 | 6.1 | 11 | 13.4 | 14.6 | MNCN-HI20 | 2006 | C. Bourdeau | HG915409 | - | HG915633 | HG915480 | HG915560 |
| 7 | 7 | Spe_chardonis | 2 | Grotte de la Valette | Alet-les-Bains | France-Pyrénées-Orientales | 2.29699 | 42.99782 | 501 | 11.4 | 6.5 | 11.6 | 14.6 | 15.8 |  |  |  |  |  |  |  |  |
| 8 | 8 | Trp_nsspecie | 248 | Grotte du Majestier | Sainte-Colombe-sur-Guette | France-Pyrénées-Orientales | 2.22642 | 42.74378 | 669 | 11.5 | 6.6 | 11.6 | 14 | 15.2 | IBE-AC114 | 2009 | C. Vanderbergh | **LN849260** | - | **LN849302** | **LN849334** | **LN849344** |
| 9 | 9 | Spe_chardonis | 3 | Grotte des Gorges de l'Aude | Axat | France-Pyrénées-Orientales | 2.22354 | 42.78694 | 498 | 12.1 | 7.1 | 12.2 | 14.8 | 16 | IBE-AC172 | 2009 | C. Bourdeau | - | **LN849361** | **LN849308** | - | - |
| 10 | 10 | Per_faurai | 252 | Coves de Rialb | Queralbs | Spain-Girona | 2.17071 | 42.33589 | 1130 | 9.5 | 4.8 | 9.6 | 11.9 | 13.1 | IBE-AC101 | 2009 | J. Comas | **LN849257** | - | **LN849299** | - | - |
| 11 | 11 | Per_bofilli | 251 | Cova de L'Estret del Forn | Queralbs | Spain-Girona | 2.13566 | 42.34189 | 1727 | 6.2 | 1.5 | 6.3 | 8.6 | 9.8 |  |  |  |  |  |  |  |  |
| 12 | 12 | Per_fresnedai | 473 | Réserve naturelle d´Eyne | Eyne | France-Pyrénées-Orientales | 2.13346 | 42.43645 |  | 0.7 | -4.1 | 0.8 | 3.2 | 4.3 |  |  |  |  |  |  |  |  |
| 13 | 14 | Trp_ludovici | 7 | Grotte du Bac de Lacaune | Coudons | France-Pyrénées-Orientales | 2.12246 | 42.86303 | 932 | 9.9 | 5 | 10 | 12.5 | 13.7 | IBE-RA526 | 2009 | C. Bourdeau | - | - | HG915634 | HG915481 | HG915561 |
| 14 | 15 | Trc_mestrei | 7 | Grotte du Bac de Lacaune | Coudons | France-Pyrénées-Orientales | 2.12246 | 42.86303 | 932 | 9.9 | 5 | 10 | 12.5 | 13.7 | IBE-AC169 | 2009 | C. Bourdeau | **LN849263** | HF912604/HF912541 | - | HF912576 | - |
| 15 | 16 | Spe_curvipes | 7 | Grotte du Bac de Lacaune | Coudons | France-Pyrénées-Orientales | 2.12246 | 42.86303 | 932 | 9.9 | 5 | 10 | 12.5 | 13.7 | IBE-AC168 | 2009 | C. Bourdeau | - | HG915700 | HG915620 | HG915471 | - |
| 16 | 17 | Per_bofilli | 250 | Coves Encantades | Queralbs | Spain-Girona | 2.11409 | 42.36097 | 2145 | 3.8 | -1 | 3.9 | 6.2 | 7.4 | NHM-IRC40 | 2001 | F. Fadrique | GU356877 | GU356773 | GU356825 | GU356924 | GU356967 |
| 17 | 18 | Tro_kiesenwetteri | 295 | Avenc Carbonera | Matadepera | Spain-Barcelona | 2.03603 | 41.63716 | 548 | 13.6 | 9.1 | 13.7 | 15.9 | 16.9 |  |  |  |  |  |  |  |  |
| 18 | 19 | Tro_kiesenwetteri | 318 | Cova S. Agnes | Matadepera | Spain-Barcelona | 2.01582 | 41.64733 | 917 | 11.5 | 6.9 | 11.5 | 13.7 | 14.8 | IBE-VR14 | 2010 | V. Rizzo | HF912467 | HF912624/HF912558 | HF912521 | - | - |
| 19 | 20 | Spe_curvipes | 5 | Grotte de l'Homme Mort | Rivel | France-Pyrénées-Orientales | 1.99531 | 42.90594 | 824 | 10.4 | 5.5 | 10.5 | 12.9 | 14.2 | MNCN-HI24 | 2006 | C. Bourdeau | **LN849285** | - | **LN849318** | **LN849339** | - |
| 20 | 21 | Spe_proserpinae | 5 | Grotte de l'Homme Mort | Rivel | France-Pyrénées-Orientales | 1.99531 | 42.90594 | 824 | 10.4 | 5.5 | 10.5 | 12.9 | 14.2 | MNCN-HI25 | 2006 | C. Bourdeau | **LN849286** | **LN849376** | - | - | - |
| 21 | 22 | Spe_curvipes | 6 | Trou du Vent de Pedrous | Bélesta | France-Ariège | 1.98058 | 42.88122 | 911 | 9.9 | 5 | 10 | 12.5 | 13.7 | IBE-AC117 | 2009 | C. Vanderbergh | **LN849261** | - | **LN849303** | - | - |
| 22 | 23 | Trc_mestrei | 6 | Trou du Vent de Pedrous | Bélesta | France-Ariège | 1.98058 | 42.88122 | 911 | 9.9 | 5 | 10 | 12.5 | 13.7 | IBE-AC112 | 2009 | C. Vanderbergh | - | - | HF912503 | - | - |
| 23 | 24 | Tro_kiesenwetteri | 296 | Avenc del Llest | Matadepera | Spain-Barcelona | 1.97758 | 41.66201 | 872 | 11.6 | 7.1 | 11.7 | 13.9 | 15 |  |  |  |  |  |  |  |  |
| 24 | 25 | Tro_kiesenwetteri | 297 | Coves de Mura | Mura | Spain-Barcelona | 1.97428 | 41.69285 | 538 | 13.6 | 9 | 13.6 | 15.9 | 16.9 | IBE-VR11 | 2010 | V. Rizzo | HF912464 | HF912621/HF912555 | HF912518 | HF912587 | - |
| 25 | 26 | Tro_ferreri | 312 | Avenc de Can Montmany | La Palma de Cervelló | Spain-Barcelona | 1.96715 | 41.41331 | 111 | 16.2 | 11.7 | 16.3 | 18.4 | 19.4 |  |  |  |  |  |  |  |  |
| 26 | 27 | Tro_ferreri | 346 | Avenc de Can Montmany | Corbera | Spain-Barcelona | 1.96269 | 41.41637 | 152 | 15.2 | 10.7 | 15.2 | 18.2 | 19.2 | IBE-VR3 | 2009 | J. Comas | HF912480 | HF912632/HF912565 | HF912531 | - | HF912601 |
| 27 | 28 | Tro_ferreri | 373 | Avenc de la Gran Desfeta | Bruguers | Spain-Barcelona | 1.95728 | 41.31221 |  | 15.2 | 10.7 | 15.2 | 17.4 | 18.3 |  |  |  |  |  |  |  |  |
| 28 | 29 | Tro_ferreri | 375 | Avenc dels Guerrillers | Garraf | Spain-Barcelona | 1.94911 | 41.29446 | 319 | 15.2 | 10.7 | 15.2 | 17.4 | 18.3 | IBE-VR30 | 2010 | V. Rizzo | HF912481 | HF912633/HF912566 | HF912532 | HF912589 | - |
| 29 | 30 | Tro_ferreri | 399 | Avenc de la Penya Esquerada nº 3 | Vallirana | Spain-Barcelona | 1.94132 | 41.35542 |  | 14.9 | 10.4 | 15 | 16.2 | 17.2 |  |  |  |  |  |  |  |  |
| 30 | 31 | Tro_ferreri | 431 | Avenc Vermell | Begues | Spain-Barcelona | 1.94009 | 41.34740 | 398 | 14.7 | 10.2 | 14.7 | 16.9 | 17.9 |  |  |  |  |  |  |  |  |
| 31 | 32 | Tro_ferreri | 389 | Avenc del Marge del Moro | Vallirana | Spain-Barcelona | 1.93793 | 41.35243 | 526 | 14 | 9.5 | 14 | 16.2 | 17.2 |  |  |  |  |  |  |  |  |
| 32 | 33 | Tro_ferreri | 390 | Avenc Marcel | Vallirana | Spain-Barcelona | 1.93638 | 41.35182 | 526 | 14 | 9.5 | 14 | 16.2 | 17.2 |  |  |  |  |  |  |  |  |
| 33 | 34 | Tro_ferreri | 443 | Esquerda del Mas de les Fonts | Ordal | Spain-Barcelona | 1.93447 | 41.35805 | 526 | 15.4 | 10.9 | 15.4 | 16.2 | 17.2 |  |  |  |  |  |  |  |  |
| 34 | 35 | Tro_ferreri | 381 | Avenc del Karst | Vallirana | Spain-Barcelona | 1.93394 | 41.37707 |  | 15.7 | 11.2 | 15.7 | 17.9 | 18.9 |  |  |  |  |  |  |  |  |
| 35 | 36 | Tro_ferreri | 416 | Avenc de la Sivinota | Gavà | Spain-Barcelona | 1.93273 | 41.28872 | 420 | 14.6 | 10.2 | 14.7 | 16.8 | 17.8 |  |  |  |  |  |  |  |  |
| 36 | 37 | Tro_ferreri | 342 | Avenc del Camí (= Avenc de les Bombes) | Vallirana | Spain-Barcelona | 1.93222 | 41.39368 |  | 15.2 | 10.7 | 15.2 | 17.4 | 18.4 |  |  |  |  |  |  |  |  |
| 37 | 38 | Spe_curvipes | 4 | Grotte Ludax | Bélesta | France-Ariège | 1.93099 | 42.88456 | 744 | 10.7 | 5.8 | 10.9 | 13.3 | 14.6 | IBE-AF201 | 2008 | C. Bourdeau & A. Faille | **LN849269** | - | **LN849312** | - | - |
| 38 | 39 | Tro_ferreri | 424 | Avenc de les Terradelles | Begues | Spain-Barcelona | 1.92963 | 41.29641 | 397 | 14.7 | 10.3 | 14.8 | 17 | 17.9 |  |  |  |  |  |  |  |  |
| 39 | 40 | Tro_ferreri | 352 | Avenc Clos | Begues | Spain-Barcelona | 1.92932 | 41.30740 | 466 | 14.3 | 9.9 | 14.4 | 16.5 | 17.5 |  |  |  |  |  |  |  |  |
| 40 | 41 | Tro_ferreri | 400 | Avenc de la Pepi | Gavà | Spain-Barcelona | 1.92887 | 41.28945 | 420 | 14.6 | 10.2 | 14.7 | 16.8 | 17.8 |  |  |  |  |  |  |  |  |
| 41 | 42 | Tro_ferreri | 437 | Cova de la Fou Montaner | Vallirana | Spain-Barcelona | 1.92793 | 41.39033 |  | 15.6 | 11.1 | 15.6 | 17.8 | 18.8 |  |  |  |  |  |  |  |  |
| 42 | 43 | Tro_ferreri | 367 | Avenc de Fou Muntaner | Vallirana | Spain-Barcelona | 1.92793 | 41.39033 |  | 15.6 | 11.1 | 15.6 | 17.8 | 18.8 |  |  |  |  |  |  |  |  |
| 43 | 44 | Tro_ferreri | 391 | Avenc del Mas Trabal | Begues | Spain-Barcelona | 1.92718 | 41.30991 | 454 | 14.4 | 10 | 14.5 | 16.6 | 17.6 |  |  |  |  |  |  |  |  |
| 44 | 45 | Tro_ferreri | 330 | Avenc de l'Arbós | Sitges | Spain-Barcelona | 1.92658 | 41.28502 | 420 | 14.6 | 10.2 | 14.7 | 16.8 | 17.8 |  |  |  |  |  |  |  |  |
| 45 | 46 | Tro_ferreri | 379 | Avenc d'en Joan Marquès | Vallirana | Spain-Barcelona | 1.92582 | 41.34996 | 495 | 13.9 | 9.4 | 13.9 | 16.3 | 17.3 |  |  |  |  |  |  |  |  |
| 46 | 47 | Tro_ferreri | 360 | Avenc de l'Esberla | Vallirana | Spain-Barcelona | 1.92501 | 41.39574 |  | 15.2 | 10.7 | 15.2 | 17.4 | 18.4 |  |  |  |  |  |  |  |  |
| 47 | 48 | Tro_ferreri | 427 | Avenc de la Troneda | Begues | Spain-Barcelona | 1.92364 | 41.30806 |  | 14.2 | 9.8 | 14.2 | 16.5 | 17.4 |  |  |  |  |  |  |  |  |
| 48 | 49 | Tro_ferreri | 429 | Avenc de les Valls | Begues | Spain-Barcelona | 1.92269 | 41.31419 | 489 | 14.2 | 9.8 | 14.2 | 16.4 | 17.4 |  |  |  |  |  |  |  |  |
| 49 | 50 | Tro_ferreri | 406 | Avenc Pomar | Vallirana | Spain-Barcelona | 1.92251 | 41.37032 | 422 | 14.6 | 10.1 | 14.6 | 16.8 | 17.8 |  |  |  |  |  |  |  |  |
| 50 | 51 | Tro_ferreri | 410 | Avenc del Puigmoltó | Begues | Spain-Barcelona | 1.92217 | 41.31318 | 489 | 14.2 | 9.8 | 14.2 | 16.4 | 17.4 |  |  |  |  |  |  |  |  |
| 51 | 52 | Tro_ferreri | 331 | Avenc de l'Arcada Petita | Gavà | Spain-Barcelona | 1.92214 | 41.29141 | 495 | 14.2 | 9.8 | 14.2 | 16.4 | 17.3 |  |  |  |  |  |  |  |  |
| 52 | 53 | Tro_ferreri | 338 | Avenc dels Bessons | Sitges | Spain-Barcelona | 1.92087 | 41.28062 | 324 | 15.2 | 10.7 | 15.2 | 17.4 | 18.3 |  |  |  |  |  |  |  |  |
| 53 | 54 | Tro_ferreri | 417 | Avenc de Sant Roc Gran | Begues | Spain-Barcelona | 1.92037 | 41.31329 |  | 14.2 | 9.8 | 14.2 | 16.4 | 17.4 | MNCN-AI1065 | 2006 | J. Fresneda | HF912496 | HF912612/HF912547 | HF912509 | HF912582 | HF912594 |
| 54 | 55 | Tro_ferreri | 418 | Avenc de Sant Roc Petit | Begues | Spain-Barcelona | 1.92031 | 41.31315 |  | 14.2 | 9.8 | 14.2 | 16.4 | 17.4 |  |  |  |  |  |  |  |  |
| 55 | 56 | Tro_ferreri | 329 | Avenc de l'Abat Escarré | Vallirana | Spain-Barcelona | 1.92015 | 41.36091 | 453 | 14.4 | 9.9 | 14.4 | 16.6 | 17.6 |  |  |  |  |  |  |  |  |
| 56 | 57 | Tro_ferreri | 407 | Avenc d'en Pompeu Fabra | Vallirana | Spain-Barcelona | 1.91969 | 41.36011 | 453 | 14.4 | 9.9 | 14.4 | 16.6 | 17.6 |  |  |  |  |  |  |  |  |
| 57 | 58 | Tro_ferreri | 411 | Avenc d'en Ramón Valls | Sitges | Spain-Barcelona | 1.91935 | 41.28078 | 324 | 15.2 | 10.7 | 15.2 | 17.4 | 18.3 |  |  |  |  |  |  |  |  |
| 58 | 59 | Tro_ferreri | 422 | Avenc T-38 | Vallirana | Spain-Barcelona | 1.91884 | 41.35906 | 453 | 14.4 | 9.9 | 14.4 | 16.6 | 17.6 |  |  |  |  |  |  |  |  |
| 59 | 60 | Tro_ferreri | 348 | Avenc de Carles Selike | Garraf | Spain-Barcelona | 1.91880 | 41.29308 | 491 | 14.2 | 9.8 | 14.2 | 16.4 | 17.4 |  |  |  |  |  |  |  |  |
| 60 | 61 | Tro_ferreri | 370 | Avenc G.I.E.S.C. | Begues | Spain-Barcelona | 1.91765 | 41.29917 | 491 | 14.2 | 9.8 | 14.2 | 16.4 | 17.4 |  |  |  |  |  |  |  |  |
| 61 | 63 | Tro_ferreri | 423 | Avenc del Tell | Sitges | Spain-Barcelona | 1.91663 | 41.28735 | 525 | 14.2 | 9.7 | 14.2 | 16.2 | 17.2 |  |  |  |  |  |  |  |  |
| 62 | 64 | Tro_ferreri | 432 | Avenc de Vinya Tita | Vallirana | Spain-Barcelona | 1.91660 | 41.35310 |  | 14.3 | 9.8 | 14.4 | 16.2 | 17.2 |  |  |  |  |  |  |  |  |
| 63 | 65 | Tro_ferreri | 368 | Avenc de la Funiosa | Begues | Spain-Barcelona | 1.91591 | 41.32359 |  | 14.7 | 10.2 | 14.7 | 16.9 | 17.9 |  |  |  |  |  |  |  |  |
| 64 | 66 | Tro_ferreri | 376 | Avenc de la Hanna Svacbroc | Sitges | Spain-Barcelona | 1.91556 | 41.28744 | 525 | 14 | 9.6 | 14.1 | 16.2 | 17.2 |  |  |  |  |  |  |  |  |
| 65 | 67 | Tro_ferreri | 369 | Avenc Geltrú | Garraf | Spain-Barcelona | 1.91492 | 41.26808 | 321 | 15.2 | 10.7 | 15.2 | 17.4 | 18.4 |  |  |  |  |  |  |  |  |
| 66 | 68 | Tro_ferreri | 377 | Avenc de l'Infern | Begues | Spain-Barcelona | 1.91479 | 41.29486 | 537 | 13.9 | 9.5 | 14 | 16.1 | 17.1 |  |  |  |  |  |  |  |  |
| 67 | 69 | Tro_ferreri | 339 | Avenc del Bufí | Begues | Spain-Barcelona | 1.91479 | 41.29486 | 537 | 13.9 | 9.5 | 14 | 16.1 | 17.1 |  |  |  |  |  |  |  |  |
| 68 | 70 | Tro_ferreri | 359 | Avenc de l'Emili Sabaté | Sitges | Spain-Barcelona | 1.91458 | 41.28032 | 398 | 14.8 | 10.3 | 14.8 | 17 | 17.9 |  |  |  |  |  |  |  |  |
| 69 | 71 | Tro_ferreri | 337 | Avenc de Benjamí Digón | Sitges | Spain-Barcelona | 1.91458 | 41.28777 | 525 | 14 | 9.6 | 14.1 | 16.2 | 17.2 |  |  |  |  |  |  |  |  |
| 70 | 72 | Tro_ferreri | 428 | Avenc dels Trons | Sitges | Spain-Barcelona | 1.91415 | 41.28777 | 525 | 14 | 9.6 | 14.1 | 16.2 | 17.2 |  |  |  |  |  |  |  |  |
| 71 | 73 | Tro_ferreri | 332 | Avenc de l'Asensio | Begues | Spain-Barcelona | 1.91352 | 41.28696 | 525 | 14 | 9.6 | 14.1 | 16.2 | 17.2 |  |  |  |  |  |  |  |  |
| 72 | 74 | Tro_ferreri | 356 | Avenc del Cuc | sitges | Spain-Barcelona | 1.91319 | 41.28716 | 525 | 14 | 9.6 | 14.1 | 16.2 | 17.2 |  |  |  |  |  |  |  |  |
| 73 | 75 | Tro_ferreri | 387 | Avenc d'en Lluís Solà | Begues | Spain-Barcelona | 1.91261 | 41.28711 | 525 | 14 | 9.6 | 14.1 | 16.2 | 17.2 |  |  |  |  |  |  |  |  |
| 74 | 76 | Tro_ferreri | 404 | Avenc de la Pleta | Sitges | Spain-Barcelona | 1.91251 | 41.27204 | 321 | 15.2 | 10.7 | 15.2 | 17.4 | 18.4 |  |  |  |  |  |  |  |  |
| 75 | 77 | Tro_ferreri | 384 | Avenc dels Llambrics | Begues | Spain-Barcelona | 1.91237 | 41.28771 | 525 | 14 | 9.6 | 14.1 | 16.2 | 17.2 |  |  |  |  |  |  |  |  |
| 76 | 78 | Tro_ferreri | 392 | Avenc d'en Morgan i Comas | Sitges | Spain-Barcelona | 1.91237 | 41.27203 | 321 | 15.2 | 10.7 | 15.2 | 17.4 | 18.4 |  |  |  |  |  |  |  |  |
| 77 | 79 | Tro_ferreri | 344 | Avenc de Campgràs | Garraf | Spain-Barcelona | 1.91220 | 41.28608 | 525 | 14 | 9.6 | 14.1 | 16.2 | 17.2 |  |  |  |  |  |  |  |  |
| 78 | 80 | Tro_ferreri | 372 | Avenc del Ginebró | Begues | Spain-Barcelona | 1.91102 | 41.30332 | 452 | 14.4 | 10 | 14.5 | 16.6 | 17.6 |  |  |  |  |  |  |  |  |
| 79 | 81 | Tro_ferreri | 401 | Avenc d'en Pere | Begues | Spain-Barcelona | 1.91102 | 41.29974 | 537 | 14.4 | 10 | 14.5 | 16.1 | 17.1 |  |  |  |  |  |  |  |  |
| 80 | 82 | Tro_ferreri | 353 | Avenc de Coll Verdaguer | Vallirana | Spain-Barcelona | 1.91095 | 41.39321 | 352 | 14.9 | 10.5 | 15 | 17.2 | 18.2 | IBE-VR35 | 2010 | V. Rizzo | HF912486 | - | - | - | - |
| 81 | 83 | Tro_ferreri | 426 | Avenc dels Tres | Begues | Spain-Barcelona | 1.91036 | 41.29278 | 537 | 13.9 | 9.5 | 14 | 16.1 | 17.1 |  |  |  |  |  |  |  |  |
| 82 | 84 | Tro_ferreri | 358 | Avenc de la Discòrdia | Begues | Spain-Barcelona | 1.91030 | 41.29299 | 537 | 13.9 | 9.5 | 14 | 16.1 | 17.1 |  |  |  |  |  |  |  |  |
| 83 | 85 | Tro_ferreri | 385 | Avenc del Llamp | Begues | Spain-Barcelona | 1.91019 | 41.28308 | 398 | 14 | 9.6 | 14.1 | 17 | 17.9 |  |  |  |  |  |  |  |  |
| 84 | 86 | Tro_ferreri | 419 | Avenc de Sant Cristòfol | Begues | Spain-Barcelona | 1.91003 | 41.29554 |  | 13.9 | 9.5 | 14 | 16.1 | 17.1 |  |  |  |  |  |  |  |  |
| 85 | 87 | Tro_ferreri | 394 | Avenc del Parpal | Begues | Spain-Barcelona | 1.90991 | 41.29419 | 537 | 13.9 | 9.5 | 14 | 16.1 | 17.1 |  |  |  |  |  |  |  |  |
| 86 | 88 | Tro_ferreri | 388 | Avenc del Mall | Begues | Spain-Barcelona | 1.90872 | 41.28979 | 525 | 14 | 9.6 | 14.1 | 16.2 | 17.2 |  |  |  |  |  |  |  |  |
| 87 | 89 | Tro_ferreri | 341 | Avenc del Bruc | Garraf | Spain-Barcelona | 1.90852 | 41.29946 | 537 | 13.9 | 9.5 | 14 | 16.1 | 17.1 |  |  |  |  |  |  |  |  |
| 88 | 90 | Tro_ferreri | 396 | Avenc del Passant | Begues | Spain-Barcelona | 1.90796 | 41.28419 | 497 | 14 | 9.6 | 14.1 | 16.4 | 17.3 |  |  |  |  |  |  |  |  |
| 89 | 91 | Tro_ferreri | 347 | Avenc de Can Sadurní | Begues | Spain-Barcelona | 1.90615 | 41.34894 | 435 | 14.5 | 10 | 14.5 | 16.7 | 17.7 |  |  |  |  |  |  |  |  |
| 90 | 92 | Tro_ferreri | 436 | Cova de Can Sadurní | Begues | Spain-Barcelona | 1.90544 | 41.34318 |  | 14.5 | 10 | 14.5 | 16.7 | 17.7 |  |  |  |  |  |  |  |  |
| 91 | 93 | Tro_ferreri | 435 | Cova/Avenc del Pla de Comes | Cervelló | Spain-Barcelona | 1.90531 | 41.39583 | 412 | 14.6 | 10.1 | 14.6 | 16.8 | 17.8 |  |  |  |  |  |  |  |  |
| 92 | 94 | Tro_ferreri | 403 | Avenc del Pla de les Bassioles | Vallirana | Spain-Barcelona | 1.90201 | 41.37384 |  | 15.2 | 10.7 | 15.2 | 17.4 | 18.4 |  |  |  |  |  |  |  |  |
| 93 | 95 | Tro_ferreri | 351 | Avenc Clar nº 2 | Cervelló | Spain-Barcelona | 1.90165 | 41.39390 | 412 | 14.6 | 10.1 | 14.6 | 16.8 | 17.8 |  |  |  |  |  |  |  |  |
| 94 | 96 | Tro_ferreri | 333 | Avenc dels Azimuts | Cervelló | Spain-Barcelona | 1.89741 | 41.39669 | 404 | 14.6 | 10.2 | 14.7 | 16.8 | 17.8 |  |  |  |  |  |  |  |  |
| 95 | 97 | Tro_ferreri | 340 | Avenc Bonic | Ordal | Spain-Barcelona | 1.89247 | 41.39560 | 404 | 14.6 | 10.2 | 14.7 | 16.8 | 17.8 |  |  |  |  |  |  |  |  |
| 96 | 98 | Tro_ferreri | 374 | Avenc de la Grèvola | Begues | Spain-Barcelona | 1.89009 | 41.31978 | 480 | 14.2 | 9.8 | 14.3 | 16.4 | 17.4 |  |  |  |  |  |  |  |  |
| 97 | 99 | Tro_ferreri | 405 | Avenc de la Plomada | Cervelló | Spain-Barcelona | 1.88820 | 41.39351 | 446 | 14.4 | 9.9 | 14.4 | 16.6 | 17.6 |  |  |  |  |  |  |  |  |
| 98 | 100 | Tro_ferreri | 412 | Avenc d'en Roca | Ordal | Spain-Barcelona | 1.88514 | 41.40196 | 524 | 13.9 | 9.4 | 14 | 16.1 | 17.1 |  |  |  |  |  |  |  |  |
| 99 | 101 | Tro_ferreri | 386 | Avenc del Llorer | Begues | Spain-Barcelona | 1.88356 | 41.31481 | 538 | 13.9 | 9.4 | 13.9 | 16.1 | 17.1 |  |  |  |  |  |  |  |  |
| 100 | 102 | Tro_ferreri | 398 | Avenc de la Penya Blanca | Begues | Spain-Barcelona | 1.88316 | 41.32391 | 439 | 14.2 | 9.8 | 14.3 | 16.6 | 17.6 |  |  |  |  |  |  |  |  |
| 101 | 103 | Tro_kiesenwetteri | 292 | Avenc Sant Salvador | Collbatò | Spain-Barcelona | 1.88261 | 41.57527 | 329 | 14.8 | 10.3 | 14.9 | 17.1 | 18.1 |  |  |  |  |  |  |  |  |
| 102 | 104 | Tro_ferreri | 357 | Avenc de la Cuneta | Olesa de Bonesvalls | Spain-Barcelona | 1.88232 | 41.36149 | 415 | 14.6 | 10.1 | 14.6 | 16.8 | 17.8 |  |  |  |  |  |  |  |  |
| 103 | 105 | Tro_ferreri | 393 | Avenc Nou | Cervelló | Spain-Barcelona | 1.87906 | 41.38957 | 459 | 14.3 | 9.8 | 14.3 | 16.5 | 17.5 |  |  |  |  |  |  |  |  |
| 104 | 106 | Tro_ferreri | 354 | Avenc del Colom (= Geòlegs) | Vallirana | Spain-Barcelona | 1.87830 | 41.38054 | 455 | 14.3 | 9.9 | 14.4 | 16.5 | 17.6 |  |  |  |  |  |  |  |  |
| 105 | 107 | Tro_kiesenwetteri | 268 | Avenc de Montserrat/Clast/ | Esparreguera | Spain-Barcelona | 1.87747 | 41.57381 | 172 | 15.7 | 11.1 | 15.7 | 17.9 | 18.9 | IBE-VR13 | 2010 | V. Rizzo & J. Comas | HF912466 | HF912623/HF912557 | HF912520 | - | - |
| 106 | 108 | Tro_ferreri | 414 | Avenc del Sellerès | Begues | Spain-Barcelona | 1.87741 | 41.34168 | 508 | 14.1 | 9.6 | 14.1 | 16.3 | 17.3 |  |  |  |  |  |  |  |  |
| 107 | 109 | Tro_ferreri | 345 | Avenc de Can Jaques | Begues | Spain-Barcelona | 1.87325 | 41.30362 | 402 | 14.7 | 10.2 | 14.7 | 16.9 | 17.9 |  |  |  |  |  |  |  |  |
| 108 | 110 | Tro_ferreri | 363 | Avenc dels Esquirols | Vallirana | Spain-Barcelona | 1.87130 | 41.38244 | 419 | 14.5 | 10.1 | 14.6 | 16.8 | 17.8 |  |  |  |  |  |  |  |  |
| 109 | 111 | Tro_ferreri | 421 | Avenc Serrano-Arbonés | Begues | Spain-Barcelona | 1.87127 | 41.30262 | 402 | 14.7 | 10.2 | 14.7 | 16.9 | 17.9 |  |  |  |  |  |  |  |  |
| 110 | 112 | Tro_ferreri | 395 | Avenc d'en Parrilla | Olesa de Bonesvalls | Spain-Barcelona | 1.86949 | 41.34853 | 435 | 14.4 | 10 | 14.5 | 16.7 | 17.7 |  |  |  |  |  |  |  |  |
| 111 | 113 | Tro_ferreri | 366 | Avenc de Font i Sagué | Ordal | Spain-Barcelona | 1.86840 | 41.37850 | 419 | 14.5 | 10.1 | 14.6 | 16.8 | 17.8 |  |  |  |  |  |  |  |  |
| 112 | 114 | Tro_ferreri | 425 | Avenc dels Topògrafs | Ordal | Spain-Barcelona | 1.86832 | 41.37863 | 419 | 14.5 | 10.1 | 14.6 | 16.8 | 17.8 |  |  |  |  |  |  |  |  |
| 113 | 115 | Tro_ferreri | 420 | Avenc de Sant Marçal | Olesa de Bonesvalls | Spain-Barcelona | 1.86744 | 41.34926 | 435 | 14.4 | 10 | 14.5 | 16.7 | 17.7 |  |  |  |  |  |  |  |  |
| 114 | 116 | Tro_ferreri | 336 | Avenc de la Bardissa | Ordal | Spain-Barcelona | 1.86257 | 41.39152 | 499 | 14.3 | 9.9 | 14.4 | 16.3 | 17.3 | IBE-AF172 | 2009 | J. Comas | HF912457 | HF912610 | - | - | - |
| 115 | 117 | Tro_kiesenwetteri | 324 | Cova del Petrecò | Esparreguera | Spain-Barcelona | 1.86253 | 41.56049 | 304 | 15 | 10.5 | 15 | 17.2 | 18.3 | IBE-VR16 | 2010 | V. Rizzo & J. Comas | HF912469 | HF912626/HF912560 | HF912523 | - | - |
| 116 | 118 | Tro_ferreri | 380 | Avenc d'en Jordi Verdiell | Ordal | Spain-Barcelona | 1.86237 | 41.38322 | 466 | 14.1 | 9.6 | 14.1 | 16.5 | 17.5 |  |  |  |  |  |  |  |  |
| 117 | 120 | Tro_ferreri | 365 | Avenc de la Ferla | Begues | Spain-Barcelona | 1.86118 | 41.31944 | 401 | 14.6 | 10.2 | 14.7 | 16.8 | 17.8 |  |  |  |  |  |  |  |  |
| 118 | 121 | Tro_ferreri | 409 | Avenc d'en Prat de la Riba | Olivella | Spain-Barcelona | 1.86046 | 41.29587 | 260 | 15.4 | 11 | 15.5 | 17.6 | 18.6 |  |  |  |  |  |  |  |  |
| 119 | 122 | Tro_ferreri | 371 | Avenc d'en Gori | Olesa de Bonesvalls | Spain-Barcelona | 1.85929 | 41.35206 | 365 | 14.8 | 10.4 | 14.9 | 17.1 | 18.1 |  |  |  |  |  |  |  |  |
| 120 | 123 | Tro_ferreri | 408 | Avenc del Pont de l'Escalat | Olesa de Bonesvalls | Spain-Barcelona | 1.85433 | 41.34702 | 298 | 15.2 | 10.7 | 15.3 | 17.4 | 18.4 |  |  |  |  |  |  |  |  |
| 121 | 124 | Tro_ferreri | 355 | Avenc del Corral Nou | Begues | Spain-Barcelona | 1.85401 | 41.30310 | 270 | 15.4 | 10.9 | 15.4 | 17.6 | 18.6 | IBE-VR26 | 2010 | V. Rizzo | HF912477 | HF912631/HF912564 | HF912530 | HF912588 | - |
| 122 | 125 | Tro_ferreri | 383 | Avenc de la Llínia | Ordal | Spain-Barcelona | 1.85382 | 41.37941 | 522 | 13.9 | 9.5 | 14 | 16.2 | 17.1 |  |  |  |  |  |  |  |  |
| 123 | 126 | Tro_ferreri | 335 | Avenc de les Banderes | Begues | Spain-Barcelona | 1.85269 | 41.32064 | 470 | 14.2 | 9.8 | 14.3 | 16.4 | 17.4 |  |  |  |  |  |  |  |  |
| 124 | 127 | Tro_ferreri | 361 | Avenc de l'Esquerrà | Olesa de Bonesvalls | Spain-Barcelona | 1.83917 | 41.32315 |  | 14.8 | 10.3 | 14.8 | 17 | 18 |  |  |  |  |  |  |  |  |
| 125 | 128 | Tro_ferreri | 440 | Cova d'Ordal (= Avenc Brut) | Ordal | Spain-Barcelona | 1.83506 | 41.40551 | 495 | 14.1 | 9.6 | 14.1 | 16.3 | 17.3 |  |  |  |  |  |  |  |  |
| 126 | 129 | Tro_kiesenwetteri | 317 | Coves de Salnitre | Collbatò | Spain-Barcelona | 1.83481 | 41.57362 | 364 | 14.6 | 10.1 | 14.7 | 16.9 | 17.9 |  |  |  |  |  |  |  |  |
| 127 | 130 | Tro_kiesenwetteri | 293 | Cova Freda | Collbatò | Spain-Barcelona | 1.83210 | 41.57398 | 410 | 14.4 | 9.9 | 14.4 | 16.7 | 17.7 | IBE-VR39 | 2010 | J. Comas | HF912490 | HF912637/HF912570 | HF912536 | - | - |
| 128 | 131 | Tro_ferreri | 364 | Avenc d'Esteles | Subirats | Spain-Barcelona | 1.83190 | 41.37278 | 467 | 14.2 | 9.8 | 14.3 | 16.4 | 17.5 |  |  |  |  |  |  |  |  |
| 129 | 132 | Cer_riberai | 260 | Cova d'en Manent | Olopte | Spain-Girona | 1.81423 | 42.38511 | 1137 | 9.2 | 4.5 | 9.3 | 11.7 | 12.9 | MNCN-AI597 | 2004 | Ph. Déliot & A. Faille | GU356862 | GU356758 | GU356812 | GU356913 | GU356952 |
| 130 | 133 | Tro_ferreri | 441 | Cova de Sant Pau | Subirats | Spain-Barcelona | 1.81034 | 41.39149 |  | 14.6 | 10.1 | 14.7 | 17.1 | 18.2 |  |  |  |  |  |  |  |  |
| 131 | 134 | Cer_riberai | 256 | Cova 1 d'Olopte | Olopte | Spain-Girona | 1.80984 | 42.38843 | 1137 | 9.2 | 4.5 | 9.3 | 11.7 | 12.9 |  |  |  |  |  |  |  |  |
| 132 | 135 | Cer_riberai | 258 | Cova 4 d'Olopte | Olopte | Spain-Girona | 1.80984 | 42.38843 | 1137 | 9.2 | 4.5 | 9.3 | 11.7 | 12.9 |  |  |  |  |  |  |  |  |
| 133 | 136 | Cer_riberai | 259 | Cova B d'Olopte | Olopte | Spain-Girona | 1.80984 | 42.38843 | 1137 | 9.2 | 4.5 | 9.3 | 11.7 | 12.9 | NHM-IRC36 | 2001 | J. Fresneda | **LN849290** | **LN849379** | **LN849323** | - | **LN849356** |
| 134 | 137 | Cer_riberai | 257 | Cova 2 d'Olopte | Olopte | Spain-Girona | 1.80984 | 42.38843 | 1137 | 9.2 | 4.5 | 9.3 | 11.7 | 12.9 |  |  |  |  |  |  |  |  |
| 135 | 138 | Tro_ferreri | 334 | Avenc B-2 | Subirats | Spain-Barcelona | 1.80862 | 41.38737 |  | 14.9 | 10.4 | 15 | 17.1 | 18.2 |  |  |  |  |  |  |  |  |
| 136 | 139 | Tro_ferreri | 343 | Avenc del Camp de Futbol | Subirats | Spain-Barcelona | 1.80484 | 41.38802 |  | 15.1 | 10.6 | 15.1 | 17.3 | 18.3 |  |  |  |  |  |  |  |  |
| 137 | 141 | Cer_cenarroi | 254 | Fou de Bor | Bellver de Cerdanya | Spain-Lleida | 1.80109 | 42.33853 | 1191 | 9 | 4.3 | 9.1 | 11.5 | 12.7 | IBE-RA463 | 2011 | C. Bourdeau & J. Fresneda | HG915359 | HG915662 | HG915584 | HG915434 | HG915505 |
| 138 | 142 | Tro_ferreri | 310 | Cova Miserachs | Sant Pau d'Ordal | Spain-Barcelona | 1.79447 | 41.38232 | 248 | 15.4 | 10.9 | 15.4 | 17.6 | 18.6 |  |  |  |  |  |  |  |  |
| 139 | 143 | Tro_kiesenwetteri | 294 | Pouetons de les Agulles | Collbatò | Spain-Barcelona | 1.78453 | 41.60768 | 882 | 11.6 | 7.1 | 11.7 | 13.9 | 15 |  |  |  |  |  |  |  |  |
| 140 | 144 | Cer_cenarroi | 253 | Forat de les Gralles | Bellver de Cerdanya | Spain-Lleida | 1.77553 | 42.33289 | 1406 | 7.8 | 3.1 | 7.9 | 10.3 | 11.5 |  |  |  |  |  |  |  |  |
| 141 | 145 | Ant_dispar | 243 | Grotte de l'Église catholique | Roquefixade | France-Ariège | 1.76936 | 42.93824 | 829 | 10.2 | 5.2 | 10.3 | 12.7 | 14 |  |  |  |  |  |  |  |  |
| 142 | 146 | Spe_longicornis | 9 | Grotte des Capètes | Freychenet | France-Ariège | 1.75525 | 42.88198 | 1222 | 8.2 | 3.3 | 8.3 | 10.7 | 12 | IBE-RA667 | 2009 | C. Bourdeau | HG915399 | - | HG915622 | HG915473 | HG915549 |
| 143 | 147 | Spe_longicornis | 12 | Gouffre de l'Artigue | Freychenet | France-Ariège | 1.72821 | 42.90650 | 842 | 10.1 | 5.2 | 10.2 | 12.7 | 14 | IBE-RA665 | 2009 | C. Bourdeau | **LN849291** | - | **LN849324** | **LN849341** | **LN849357** |
| 144 | 148 | Spe_piochardi | 12 | Gouffre de l'Artigue | Freychenet | France-Ariège | 1.72821 | 42.90650 | 842 | 10.1 | 5.2 | 10.2 | 12.7 | 14 |  |  |  |  |  |  |  |  |
| 145 | 149 | Tro_olerdolai | 321 | Avenc d'Olèrdola | Olèrdola | Spain-Barcelona | 1.72469 | 41.31816 | 188 | 15.8 | 11.3 | 15.8 | 17.9 | 18.8 |  |  |  |  |  |  |  |  |
| 146 | 150 | Spn_mengeli | 201 | Bofia de Sant Jaume de Montmajor | Avià | Spain-Lleida | 1.72251 | 42.05815 |  | 10.7 | 6 | 10.8 | 13 | 14.2 |  |  |  |  |  |  |  |  |
| 147 | 151 | Tro_kiesenwetteri | 319 | Cova del Pas | Castellolì | Spain-Barcelona | 1.70524 | 41.58826 | 519 | 13.6 | 9.1 | 13.7 | 15.9 | 17 | IBE-VR18 | 2010 | J. Comas & V. Rizzo | HF912471 | HF912628/HF912562 | HF912525 | - | - |
| 148 | 151 | Tro_kiesenwetteri | 319 | Cova del Pas | Castellolì | Spain-Barcelona | 1.70524 | 41.58826 | 519 | 13.6 | 9.1 | 13.7 | 15.9 | 17 | IBE-VR7 | 2009 | V. Rizzo & J. Comas | **LN849297** | HF912641/HF912574 | - | - | - |
| 149 | 152 | Spn_mengeli | 210 | Cova del Pont Quebradís | Gòsol | Spain-Lleida | 1.69714 | 42.17777 | 1166 | 9.3 | 4.6 | 9.4 | 11.7 | 12.9 |  |  |  |  |  |  |  |  |
| 150 | 153 | Spn_mengeli | 213 | Bòfia Esglevades | Navés | Spain-Lleida | 1.67833 | 42.10333 |  | 10.6 | 5.9 | 10.7 | 13 | 14.2 |  |  |  |  |  |  |  |  |
| 151 | 154 | Spn_mengeli | 220 | Avenc de Querforadat | Querforadat | Spain-Lleida | 1.64505 | 42.32547 |  | 8.3 | 3.6 | 8.4 | 10.8 | 12 |  |  |  |  |  |  |  |  |
| 152 | 155 | Spn_mengeli | 221 | Avenc de la Cabana d'en Garraba | Toloriu | Spain-Lleida | 1.63794 | 42.36077 |  | 9.1 | 4.4 | 9.2 | 11.6 | 12.8 |  |  |  |  |  |  |  |  |
| 153 | 156 | Spe_pyreneus | 17 | Grotte de Lombrives | Ussat | France-Ariège | 1.61627 | 42.82334 | 488 | 12 | 7.1 | 12.1 | 12.1 | 13.4 | MNCN-AC55 | 2006 | A. Faille | - | - | **LN849298** | - | **LN849342** |
| 154 | 157 | Ant_querilhaci | 17 | Grotte de Lombrives | Ussat | France-Ariège | 1.61627 | 42.82334 | 488 | 12 | 7.1 | 12.1 | 12.1 | 13.4 | IBE-AC130 | 2006 | A. Faille | - | - | **LN849306** | - | - |
| 155 | 158 | Spn_mengeli | 222 | Cova de les Encantades (= Cova de Vinyoles. Cova de Coll de Sé, Cova de Borgunyà) | Toloriu | Spain-Lleida | 1.61123 | 42.34848 | 1290 | 8.3 | 3.6 | 8.4 | 10.8 | 12 |  |  |  |  |  |  |  |  |
| 156 | 159 | Spn_mengeli | 208 | Cova del Rabeig | Lladurs | Spain-Lleida | 1.57944 | 42.09667 |  | 11.8 | 7.1 | 11.9 | 14.3 | 15.4 |  |  |  |  |  |  |  |  |
| 157 | 160 | Tro_jacasi | 316 | Cova de les Rondes | La Llacuna | Spain-Barcelona | 1.54667 | 41.47097 | 740 | 12.4 | 7.9 | 12.5 | 14.7 | 15.8 | IBE-VR2 | 2009 | J.M. Victoria | - | HF912629 | HF912526 | - | - |
| 158 | 160 | Tro_jacasi | 316 | Cova de les Rondes | La Llacuna | Spain-Barcelona | 1.54667 | 41.47097 | 740 | 12.4 | 7.9 | 12.5 | 14.7 | 15.8 | IBE-VR22 | 2010 | J. Comas & V. Rizzo | HF912474 | - | HF912528 | - | - |
| 159 | 161 | Ant_querilhaci | 467 | Grotte de Siech | Saurat | France-Ariège | 1.54560 | 42.88270 |  | 11 | 6 | 11.1 | 13.5 | 14.8 |  |  |  |  |  |  |  |  |
| 160 | 162 | Tro_jacasi | 291 | Avenc Viumala | La Llacuna | Spain-Barcelona | 1.53671 | 41.46589 | 722 | 12.5 | 8 | 12.6 | 14.8 | 15.9 |  |  |  |  |  |  |  |  |
| 161 | 163 | Tro_schibii | 269 | Avenc de la Solana | Querol | Spain-Barcelona | 1.53513 | 41.45500 | 796 | 12.1 | 7.6 | 12.2 | 14.4 | 15.4 |  |  |  |  |  |  |  |  |
| 162 | 164 | Spn_mengeli | 216 | Avenc de la Canaleta | Odén | Spain-Lleida | 1.52583 | 42.11389 |  | 9.8 | 5.1 | 9.9 | 12.3 | 13.5 |  |  |  |  |  |  |  |  |
| 163 | 165 | Spn_mengeli | 207 | Cova Edes | Canalda | Spain-Lleida | 1.52028 | 42.13333 |  | 7.7 | 3 | 7.8 | 11.3 | 12.5 |  |  |  |  |  |  |  |  |
| 164 | 166 | Spn_mengeli | 206 | Avenc Costa Bòfia | Canalda | Spain-Lleida | 1.50917 | 42.12167 |  | 9.2 | 4.5 | 9.3 | 11.6 | 12.8 |  |  |  |  |  |  |  |  |
| 165 | 167 | Spe_abeillei | 21 | Grotte de Siech | Saurat | France-Ariège | 1.49883 | 42.88371 | 1012 | 9.1 | 4.2 | 9.2 | 11.7 | 13 | IBE-RA758 | 2010 | C. Vanderbergh | - | **LN849365** | **LN849325** | - | **LN849358** |
| 166 | 168 | Tro_elongatus | 271 | Avenc de l'Arlà | Albinyana | Spain-Barcelona | 1.49097 | 41.25532 | 217 | 15.4 | 10.9 | 15.4 | 17.6 | 18.6 |  |  |  |  |  |  |  |  |
| 167 | 169 | Tro_elongatus | 508 | Avenc de la Plana d'Ancosa | La Llacuna | Spain-Barcelona | 1.48962 | 41.44670 |  | 12.4 | 7.9 | 12.5 | 14.8 | 15.8 | IBE-VR1 | 2009 | J. Comas | HF912462 | HF912619/HF912553 | HF912516 | - | - |
| 168 | 170 | Tro_elongatus | 488 | Avenc Victoria | Montral | Spain-Tarragona | 1.48853 | 41.35022 |  | 12.7 | 8.2 | 12.7 | 14.9 | 16 |  |  |  |  |  |  |  |  |
| 169 | 171 | Tro_elongatus | 270 | Cova de Cal Fontena | Pontons | Spain-Barcelona | 1.48732 | 41.41583 | 689 | 12.7 | 8.2 | 12.8 | 15 | 16 |  |  |  |  |  |  |  |  |
| 170 | 172 | Spe_longicornis | 11 | Grotte de Pigaï | Aigües Juntes | France-Ariège | 1.48724 | 43.05400 | 441 | 11.8 | 6.8 | 12 | 14.4 | 15.8 | MNCN-AI531 | 2004 | A. Faille | HG915400 | HG915702 | HG915623 | - | HG915550 |
| 171 | 173 | Ant_dispar | 10 | Grotte Les Cloutets | Aigües Juntes | France-Ariège | 1.48192 | 43.05666 | 375 | 12.2 | 7.2 | 12.3 | 14.8 | 16.1 | MNCN-AI588 | 2004 | A. Faille | GU356851 | GU356746 | GU356803 | GU356905 | GU356942 |
| 172 | 174 | Spe_longicornis | 10 | Grotte Les Cloutets | Aigües Juntes | France-Ariège | 1.48192 | 43.05666 | 375 | 12.2 | 7.2 | 12.3 | 14.8 | 16.1 |  |  |  |  |  |  |  |  |
| 173 | 175 | Spn_mengeli | 215 | Avenc dels Encantats | Odén | Spain-Lleida | 1.47917 | 42.11917 |  | 8.8 | 4.1 | 8.9 | 11.3 | 12.5 |  |  |  |  |  |  |  |  |
| 174 | 176 | Tro_elongatus | 273 | Cova de l'Olla | Montmell | Spain-Barcelona | 1.47492 | 41.31495 | 509 | 14 | 9.5 | 14 | 16 | 17.1 | IBE-VR17 | 2010 | J. Comas & V. Rizzo | HF912470 | HF912627/HF912561 | HF912524 | - | - |
| 175 | 177 | Tro_elongatus | 272 | Cova avenc de l'Artús | Albinyana | Spain-Barcelona | 1.47485 | 41.24250 | 367 | 15 | 10.6 | 15.1 | 16.8 | 17.8 |  |  |  |  |  |  |  |  |
| 176 | 178 | Tro_elongatus | 274 | Cova de Vallmajor | Albinyana | Spain-Barcelona | 1.47463 | 41.23645 | 349 | 15.4 | 11 | 15.4 | 16.9 | 17.9 | IBE-VR15 | 2010 | V. Rizzo & J. Comas | HF912468 | HF912625/HF912559 | HF912522 | - | - |
| 177 | 179 | Spn_andorranus | 198 | Cova de la Margineda | Sant Julià de Lòria | Andorra | 1.47171 | 42.47927 | 1055 | 9.4 | 4.5 | 9.4 | 11.8 | 13.1 |  |  |  |  |  |  |  |  |
| 178 | 180 | Spn_andorranus | 197 | Cova de la Gorga de la Margineda | Sant Julià de Lòria | Andorra | 1.47171 | 42.47927 | 1055 | 9.4 | 4.5 | 9.4 | 11.8 | 13.1 |  |  |  |  |  |  |  |  |
| 179 | 181 | Ant_querilhaci | 242 | Trou du Rantou | Suc et Sentenac | France-Ariège | 1.45869 | 42.78498 | 1059 | 9 | 4.1 | 9.1 | 11.5 | 12.8 | IBE-AC118 | 2009 | C. Vanderbergh | **LN849262** | - | **LN849304** | - | - |
| 180 | 183 | Spn_mengeli | 214 | Avenc de Coll Alsina | Odén | Spain-Lleida | 1.45060 | 42.10906 |  | 9.7 | 4.9 | 9.7 | 12.1 | 13.3 |  |  |  |  |  |  |  |  |
| 181 | 184 | Spe_piochardi | 446 | Grotte de la Garosse | Unjat | France-Ariège | 1.44763 | 43.02407 |  | 11.5 | 6.5 | 11.6 | 14.1 | 15.4 | IBE-AC119 | 2009 | C. Vanderbergh | - | - | **LN849305** | **LN849335** | **LN849345** |
| 182 | 185 | Ant_dispar | 29 | Grotte de Ferrobach | Alzen | France-Ariège | 1.44521 | 42.98778 | 606 | 11.1 | 6.1 | 11.2 | 13.7 | 15 | MNCN-AI594 | 2004 | A. Faille | HE572810 | HE576711 | - | - | - |
| 183 | 186 | Spe_normandi | 29 | Grotte de Ferrobach | Alzen | France-Ariège | 1.44521 | 42.98778 | 606 | 11.1 | 6.1 | 11.2 | 13.7 | 15 | MNCN-AI532 | 2004 | A. Faille | HG915402 | HG915704 | HG915625 | HG915475 | HG915552 |
| 184 | 187 | Tro_elongatus | 305 | Avenc d'Ancosa | Igualada | Spain-Barcelona | 1.44299 | 41.37500 | 677 | 12.2 | 7.7 | 12.2 | 15.1 | 16.1 |  |  |  |  |  |  |  |  |
| 185 | 188 | Spn_mengeli | 205 | Cova de Ca l’Espunyes | Cambrils | Spain-Lleida | 1.43733 | 42.15189 | 1695 | 6.2 | 1.5 | 6.3 | 8.7 | 9.9 |  |  |  |  |  |  |  |  |
| 186 | 189 | Tro_subilsi | 303 | Grallera de Cambrils | Llinars-Cambrils | Spain-Lleida | 1.43733 | 42.15189 | 1770 | 6.2 | 1.5 | 6.3 | 8.7 | 9.9 |  |  |  |  |  |  |  |  |
| 187 | 190 | Spn_mengeli | 224 | Avenc de Serra Garriga | Odén | Spain-Lleida | 1.43460 | 42.11039 |  | 10.9 | 6.2 | 11 | 13.3 | 14.5 |  |  |  |  |  |  |  |  |
| 188 | 191 | Tro_elongatus | 307 | Avenc de Pinyarets | El Montmell | Spain-Barcelona | 1.43372 | 41.31495 | 499 | 13.8 | 9.3 | 13.8 | 16.1 | 17.1 | IBE-VR38 | 2010 | J.M. Victoria | HF912489 | HF912636/HF912569 | HF912535 | - | - |
| 189 | 192 | Spn_mengeli | 203 | Baborell del Pic del Montsec | Tora de Tost | Spain-Lleida | 1.43190 | 42.26045 |  | 7.1 | 2.3 | 7.2 | 9.6 | 10.8 |  |  |  |  |  |  |  |  |
| 190 | 193 | Spe_colluvii | 469 | Bois de Candail (S109) | Massat | France-Ariège | 1.42500 | 42.89360 |  | 8.6 | 3.6 | 8.7 | 11.5 | 12.8 |  |  |  |  |  |  |  |  |
| 191 | 194 | Spe_colluvii | 472 | Coume Longe (SE Bernes peak, road bt Carol and Goutest) | Massat | France-Ariège | 1.41650 | 42.84780 |  | 7.5 | 2.6 | 7.6 | 10.9 | 12.2 |  |  |  |  |  |  |  |  |
| 192 | 195 | Spe_colluvii | 471 | La Plagne (SE Bernes peak, road bt Carol and Goutest) | Massat | France-Ariège | 1.41520 | 42.84810 |  | 8.3 | 3.4 | 8.4 | 10.9 | 12.2 |  |  |  |  |  |  |  |  |
| 193 | 196 | Spn_mengeli | 204 | Dolina de coll d’Arnat | Castellar de Tost | Spain-Lleida | 1.40876 | 42.25142 |  | 7.9 | 3.2 | 8 | 10.4 | 11.7 |  |  |  |  |  |  |  |  |
| 194 | 197 | Spe_chardonis | 457 | grotte de Bérac | Boussenac | France-Ariège | 1.36738 | 43.06646 | 384 | 11.9 | 6.8 | 12 | 14.7 | 16 |  |  |  |  |  |  |  |  |
| 195 | 198 | Ant_dispar | 468 | Gouffre VM1 | Le Port | France-Ariège | 1.36470 | 42.81140 |  | 6.2 | 1.3 | 6.3 | 8.8 | 10.1 |  |  |  |  |  |  |  |  |
| 196 | 199 | Spn_mengeli | 219 | Cova de les Encantades | Toloriu | Spain-Lleida | 1.36274 | 42.12814 |  | 8.6 | 3.9 | 8.7 | 11 | 12.2 |  |  |  |  |  |  |  |  |
| 197 | 200 | Spn_mengeli | 218 | Avenc del Xato | Llinars | Spain-Lleida | 1.36255 | 42.13080 | 1269 | 8.6 | 3.9 | 8.7 | 11 | 12.2 |  |  |  |  |  |  |  |  |
| 198 | 201 | Spe_longicornis | 463 | Grotte du Mas-d'Azil | Le Mas d'Azil | France-Ariège | 1.35560 | 43.06984 |  | 11.7 | 6.6 | 11.8 | 14.3 | 15.6 |  |  |  |  |  |  |  |  |
| 199 | 202 | Tro_elongatus | 306 | Cova del Garrofet | Querol | Spain-Barcelona | 1.35132 | 41.42831 | 730 | 12.4 | 7.9 | 12.5 | 14.7 | 15.8 | IBE-VR4 | 2009 | J. Comas | HF912491 | HF912638/HF912571 | HF912537 | - | HF912602 |
| 200 | 203 | Ant_dispar | 18 | Grotte de Peyrounard | Le Mas d'Azil | France-Ariège | 1.34880 | 43.07537 | 461 | 11.7 | 6.6 | 11.8 | 14.2 | 15.6 |  |  |  |  |  |  |  |  |
| 201 | 204 | Spe_stygius | 18 | Grotte de Peyrounard | Le Mas d'Azil | France-Ariège | 1.34880 | 43.07537 | 461 | 11.7 | 6.6 | 11.8 | 14.2 | 15.6 |  |  |  |  |  |  |  |  |
| 202 | 205 | Spe_abeillei | 18 | Grotte de Peyrounard | Le Mas d'Azil | France-Ariège | 1.34880 | 43.07537 | 461 | 11.7 | 6.6 | 11.8 | 14.2 | 15.6 |  |  |  |  |  |  |  |  |
| 203 | 206 | Tro_fonti | 286 | Forat de l'Infern | Les Valls d'Aguilar | Spain-Lleida | 1.34503 | 42.28169 | 885 | 10.4 | 5.7 | 10.5 | 12.9 | 14.2 |  |  |  |  |  |  |  |  |
| 204 | 207 | Spe_colluvii | 470 | Camping de La Chapelle | Massat | France-Ariège | 1.34370 | 42.88870 |  | 10.9 | 6 | 11 | 13.5 | 14.8 |  |  |  |  |  |  |  |  |
| 205 | 208 | Tro_ludovici | 320 | Forat de les Cases Noves | Organyà | Spain-Lleida | 1.34000 | 42.20316 | 595 | 12.2 | 7.4 | 12.2 | 14.6 | 15.8 |  |  |  |  |  |  |  |  |
| 206 | 209 | Tro_vinyasi | 320 | Forat de les Cases Noves | Organyà | Spain-Lleida | 1.34000 | 42.20316 | 595 | 12.2 | 7.4 | 12.2 | 14.6 | 15.8 |  |  |  |  |  |  |  |  |
| 207 | 210 | Spn_mengeli | 209 | Avenc del Pont Espia | Coll de Nargó | Spain-Lleida | 1.33210 | 42.18713 | 560 | 12.3 | 7.6 | 12.4 | 14.8 | 16 |  |  |  |  |  |  |  |  |
| 208 | 211 | Spe_stygius | 20 | Grotte de Malarnaud | Montseron | France-Ariège | 1.32923 | 43.01938 | 467 | 11.7 | 6.7 | 11.8 | 14.3 | 15.6 | NHM-RB9 |  | Ph. Déliot & A. Faille | - | **LN849385** | **LN849332** | - | - |
| 209 | 212 | Spe_zophosinus | 31 | Grotte Rieux | Massat | France-Ariège | 1.32700 | 42.89559 | 608 | 11.1 | 6.2 | 11.2 | 13.7 | 15 |  |  |  |  |  |  |  |  |
| 210 | 213 | Spe_zophosinus | 32 | Grotte du Ker | Massat | France-Ariège | 1.32590 | 42.89567 | 608 | 11.1 | 6.2 | 11.2 | 13.7 | 15 | MNCN-AI667 | 2006 | C. Bourdeau | HF912500 | HF912616/HF912551 | HF912513 | HF912584 | HF912598 |
| 211 | 214 | Spe_pyreneus | 32 | Grotte du Ker | Massat | France-Ariège | 1.32590 | 42.89567 | 608 | 11.1 | 6.2 | 11.2 | 13.7 | 15 | MNCN-AI668 | 2006 | C. Bourdeau | HG915404 | HG915705 | HG915627 | - | HG915554 |
| 212 | 215 | Tro_subilsi | 326 | Grallera de Cambrils | Cambrils | Spain-Lleida | 1.31596 | 42.05286 | 529 | 12.7 | 8 | 12.8 | 15.1 | 16.3 |  |  |  |  |  |  |  |  |
| 213 | 216 | Spe_normandi | 27 | Grotte de Rougé | Riverenert | France-Ariège | 1.29194 | 42.95222 | 877 | 9.6 | 4.6 | 9.7 | 12.2 | 13.5 | MNCN-AI528 | 2004 | A. Faille | HG915401 | HG915703 | HG915624 | HG915474 | HG915551 |
| 214 | 217 | Tro_fonti | 466 | Cova de la Font de Miravall | Miravall | Spain-Lleida | 1.28471 | 42.32230 |  | 8.5 | 3.7 | 8.6 | 11 | 12.2 |  |  |  |  |  |  |  |  |
| 215 | 218 | Tro_fonti | 315 | Cova de Guils | Guils del Cantó | Spain-Lleida | 1.28323 | 42.34566 | 1341 | 6.9 | 2.1 | 7 | 10.4 | 11.6 |  |  |  |  |  |  |  |  |
| 216 | 219 | Tro_espanoli | 309 | Cova del Traça | Fontcaldetes | Spain-Tarragona | 1.28265 | 41.40154 | 540 | 13.4 | 8.9 | 13.5 | 15.7 | 16.8 | IBE-VR23 | 2010 | F. Fadrique | HF912475 | HF912630/HF912563 | HF912529 | - | - |
| 217 | 220 | Tro_vinyasi | 327 | Avenc del Mort | Peramola | Spain-Lleida | 1.26798 | 42.07816 | 981 | 10.2 | 5.5 | 10.2 | 12.6 | 13.8 |  |  |  |  |  |  |  |  |
| 218 | 221 | Tro_fonti | 279 | Cova de l'Oli Ermì | Espaèn | Spain-Lleida | 1.26194 | 42.31980 | 1200 | 8.7 | 3.9 | 8.7 | 11.1 | 12.4 |  |  |  |  |  |  |  |  |
| 219 | 222 | Tro_fonti | 276 | Avenc de la roca del corb | Peramola | Spain-Lleida | 1.25828 | 42.07631 | 870 | 10.9 | 6.2 | 10.9 | 13.2 | 14.4 |  |  |  |  |  |  |  |  |
| 220 | 223 | Spn_mengeli | 212 | Avenc de les Bombes | Montanisell | Spain-Lleida | 1.24222 | 42.19958 |  | 8.9 | 4.2 | 9 | 11.4 | 12.7 |  |  |  |  |  |  |  |  |
| 221 | 224 | Tro_fonti | 278 | Bòfia del Tossal del Morro | Cabò | Spain-Lleida | 1.23501 | 42.21958 | 979 | 10 | 5.2 | 10.1 | 12.5 | 13.7 |  |  |  |  |  |  |  |  |
| 222 | 225 | Tro_fonti | 282 | Matella de les planes | Cabò | Spain-Lleida | 1.23332 | 42.21953 | 1151 | 10 | 5.2 | 10.1 | 11.5 | 12.7 |  |  |  |  |  |  |  |  |
| 223 | 226 | Tro_fonti | 447 | Avenc del Pla Fornesa | La Guardia d'Ares | Spain-Lleida | 1.23322 | 42.27245 |  | 7.4 | 2.7 | 7.5 | 9.6 | 10.8 | IBE-AF181 | 2009 | C. Bourdeau & A. Faille | HF912458 | - | - | - | - |
| 224 | 227 | Tro_fonti | 285 | Avenc d'en Viladrich | Peramola | Spain-Lleida | 1.22659 | 42.04651 | 989 | 10.2 | 5.5 | 10.2 | 12.6 | 13.8 |  |  |  |  |  |  |  |  |
| 225 | 228 | Tro_fonti | 280 | Cova d'ormini | Montanissel | Spain-Lleida | 1.22596 | 42.20703 | 1588 | 6.6 | 1.9 | 6.7 | 9.1 | 10.4 | NHM-IRC26 | 2001 | J. Fresneda | GU356902 | GU356801 | GU356848 | - | GU356993 |
| 226 | 229 | Tro_fonti | 277 | Avenc del Cataplanell | Coll de Nargò | Spain-Lleida | 1.22023 | 42.16627 | 1209 | 8.8 | 4 | 8.8 | 11.2 | 12.5 |  |  |  |  |  |  |  |  |
| 227 | 230 | Spe_pyreneus | 16 | Grotte de la Quére | Mérigon | France-Ariège | 1.21074 | 43.08406 | 450 | 11.6 | 6.6 | 11.7 | 14.3 | 15.6 | NHM-RB5 |  | Ph. Déliot & A. Faille | - | **LN849382** | **LN849329** | - | - |
| 228 | 231 | Par_jeanneli | 454 | Aven d´Anglade | Couflens | France-Ariège | 1.19864 | 42.73447 |  | 6.7 | 1.8 | 6.8 | 9.3 | 10.6 |  |  |  |  |  |  |  |  |
| 229 | 232 | Met_monticola | 454 | Aven d' Anglade | Couflens | France-Ariège | 1.19864 | 42.73447 |  | 6.7 | 1.8 | 6.8 | 9.3 | 10.6 |  |  |  |  |  |  |  |  |
| 230 | 233 | Pas_vandeli | 33 | Grotte de Las Souleillos | Seix | France-Ariège | 1.18530 | 42.84021 | 749 | 10.4 | 5.4 | 10.5 | 13 | 14.3 | MNCN-AI539 | 2004 | C. Bourdeau & A. Faille | GU356875 | GU356771 | HE572881 | - | HE572867 |
| 231 | 234 | Spe_stygius | 1 | Faille du Jouhandet | Seix | France-Ariège | 1.18500 | 42.84201 |  | 10.9 | 6 | 11.1 | 13.5 | 14.8 |  |  |  |  |  |  |  |  |
| 232 | 235 | Met_monticola | 1 | Faille du Jouhandet | Seix | France-Ariège | 1.18500 | 42.84201 |  | 10.9 | 6 | 11.1 | 13.5 | 14.8 |  |  |  |  |  |  |  |  |
| 233 | 236 | Spe_stygius | 14 | Grotte d'Ardet | Rogalle | France-Ariège | 1.18448 | 42.90276 | 850 | 9.8 | 4.8 | 9.9 | 12.3 | 13.7 | NHM-RB7 |  | Ph. Déliot & A. Faille | - | **LN849384** | **LN849331** | - | - |
| 234 | 237 | Spe_pyreneus | 14 | Grotte d'Ardet | Rogalle | France-Ariège | 1.18448 | 42.90276 | 850 | 9.8 | 4.8 | 9.9 | 12.3 | 13.7 | MNCN-AI535 | 2004 | C. Bourdeau | HG915403 | HG915706 | HG915626 | - | HG915553 |
| 235 | 237 | Spe_pyreneus | 14 | Grotte d'Ardet | Rogalle | France-Ariège | 1.18448 | 42.90276 | 850 | 9.8 | 4.8 | 9.9 | 12.3 | 13.7 | NHM-RB6 |  | Ph. Déliot & A. Faille | **LN849296** | **LN849383** | **LN849330** | - | - |
| 236 | 238 | Tro_fonti | 290 | Cova Palomera | Taús | Spain-Lleida | 1.17715 | 42.28587 | 1394 | 7.6 | 2.8 | 7.6 | 10.1 | 11.3 | NHM-IRC22 | 2001 | J. Fresneda | HF912501 | HF912617 | HF912514 | - | HF912599 |
| 237 | 239 | Tro_orcinus | 301 | Esquerda del Puig de Marc | La Riba | Spain-Tarragona | 1.16575 | 41.30425 | 652 | 12.8 | 8.4 | 12.9 | 15.2 | 16.3 |  |  |  |  |  |  |  |  |
| 238 | 240 | Tro_orcinus | 322 | Forat del Castell de la Formiga | Vilaverd | Spain-Tarragona | 1.16464 | 41.32191 | 455 | 13.9 | 9.4 | 14 | 16.2 | 17.3 |  |  |  |  |  |  |  |  |
| 239 | 241 | Spe_pyreneus | 13 | Grotte de Sabouche inf. | Eycheil | France-Ariège | 1.16362 | 42.96251 | 519 | 11.4 | 6.4 | 11.5 | 14 | 15.3 | IBE-RA670 | 2009 | C. Bourdeau & J. Fresneda | **LN849292** | - | - | - | - |
| 240 | 242 | Tro_fonti | 275 | Av del pla de Ribes | Abella de la Conca | Spain-Lleida | 1.16204 | 42.19052 | 1588 | 6.6 | 1.9 | 6.7 | 9.1 | 10.3 |  |  |  |  |  |  |  |  |
| 241 | 243 | Tro_orcinus | 507 | Cova Cartanyà | La Riba | Spain-Tarragona | 1.16185 | 41.31746 |  | 13.9 | 9.4 | 14 | 16.2 | 17.3 | IBE-VR20 | 2010 | F. Fadrique | HF912472 | - | HF912527 | - | - |
| 242 | 244 | Spe_normandi | 28 | Ruisseau souterrain d'Aulot | Saint Girons | France-Ariège | 1.15630 | 42.97673 | 397 | 12 | 7 | 12.1 | 14.6 | 15.9 | MNCN-AI537 | 2004 | A. Faille | **LN849271** | - | - | - | - |
| 243 | 245 | Trp_aubryi | 245 | Grotte de Esbints | Seix | France-Ariège | 1.13944 | 42.85139 |  | 9 | 4.1 | 9.1 | 11.6 | 12.9 |  |  |  |  |  |  |  |  |
| 244 | 246 | Ges_delioti | 249 | Bordes de Crues | Seix | France-Ariège | 1.13848 | 42.81434 | 1119 | 8.4 | 3.5 | 8.5 | 11 | 12.3 | MNCN-AI525 | 2004 | J. Fresneda | GU356867 | GU356763 | - | GU356916 | GU356959 |
| 245 | 247 | Tro_orcinus | 299 | Avenc de la Figuera | Montblanc | Spain-Tarragona | 1.13468 | 41.35226 | 480 | 13.8 | 9.3 | 13.8 | 16.1 | 17.2 |  |  |  |  |  |  |  |  |
| 246 | 248 | Tro_fonti | 314 | Font Mentidora | Hortoneda de la Conca | Spain-Lleida | 1.13333 | 42.23333 | 1921 | 4.2 | -0.6 | 4.3 | 7.2 | 8.5 | IBE-VR37 | 2010 | J. Fresneda, I. Ribera & V. Rizzo | HF912488 | - | HF912534 | - | - |
| 247 | 249 | Tro_orcinus | 504 | Cova Can Masiet | Reus | Spain-Tarragona | 1.12911 | 41.29975 |  | 13.8 | 9.4 | 13.9 | 14.9 | 16 | IBE-VR10 | 2010 | V. Rizzo | HF912463 | HF912620/HF912554 | HF912517 | HF912586 | - |
| 248 | 250 | Tro_orcinus | 328 | Avenc Nou de la Font Freda | Alcover | Spain-Tarragona | 1.12910 | 41.24890 | 468 | 13.9 | 9.4 | 13.9 | 16.2 | 17.3 |  |  |  |  |  |  |  |  |
| 249 | 251 | Pal_pallaresana | 236 | Cova Saverneda (= Cova del Drac) | Sort | Spain-Lleida | 1.12879 | 42.38804 | 743 | 11 | 6.2 | 11 | 13.5 | 14.8 | IBE-AF123 | 2009 | J. Fresneda, A. Faille & C. Bourdeau | HF912455 | HF912607/HF912544 | HF912505 | HF912578 | - |
| 250 | 252 | Spn_crypticola | 236 | Cova Saverneda (= Cova del Drac) | Sort | Spain-Lleida | 1.12879 | 42.38804 | 743 | 11 | 6.2 | 11 | 13.5 | 14.8 |  |  |  |  |  |  |  |  |
| 251 | 253 | Tro_fonti | 481 | Forat de Cuberes | Baix Pallars | Spain-Lleida | 1.12823 | 42.27812 |  | 6.9 | 2.1 | 6.9 | 9.4 | 10.6 |  |  |  |  |  |  |  |  |
| 252 | 254 | Tro_orcinus | 482 | La Petita de Bonretorn | L'Albiol | Spain-Tarragona | 1.12819 | 41.24756 |  | 13.9 | 9.4 | 13.9 | 16.2 | 17.3 |  |  |  |  |  |  |  |  |
| 253 | 255 | Tro_fonti | 289 | Cova del Clot de Cuberes | Baix Pallars | Spain-Lleida | 1.12812 | 42.27804 | 1519 | 6.9 | 2.1 | 6.9 | 9.4 | 10.6 |  |  |  |  |  |  |  |  |
| 254 | 256 | Spe_pyreneus | 15 | Grotte de Tourtouse | Tourtouse | France-Ariège | 1.12672 | 43.09075 | 393 | 11.9 | 6.8 | 12 | 14.5 | 15.8 | IBE-AF57 | 2008 | C. Bourdeau & A. Faille | **LN849265** | - | **LN849309** | - | - |
| 255 | 257 | Spe_stygius | 19 | Grotte de la Touasse Peyrous | Taurignan | France-Ariège | 1.11573 | 43.03923 | 436 | 11.7 | 6.7 | 11.8 | 14.3 | 15.7 |  |  |  |  |  |  |  |  |
| 256 | 258 | Spe_fagniezi | 8 | Grotte d'Aubert | Moulis | France-Ariège | 1.11527 | 42.95865 | 665 | 10.6 | 5.6 | 10.7 | 13.2 | 14.5 |  |  |  |  |  |  |  |  |
| 257 | 259 | Spe_stygius | 8 | Grotte d'Aubert | Moulis | France-Ariège | 1.11527 | 42.95865 | 665 | 10.6 | 5.6 | 10.7 | 13.2 | 14.5 | MNCN-AI529 | 2004 | A. Faille | GU356898 | GU356797 | GU356844 | - | GU356989 |
| 258 | 260 | Spe_diecki | 8 | Grotte d'Aubert | Moulis | France-Ariège | 1.11527 | 42.95865 | 665 | 10.6 | 5.6 | 10.7 | 13.2 | 14.5 | MNCN-AI536 | 2004 | A. Faille | GU356896 | GU356794 | GU356841 | GU356938 | GU356986 |
| 259 | 261 | Tro_fonti | 283 | Pou de Graells | Isona | Spain-Lleida | 1.10833 | 42.07634 | 1127 | 8.9 | 4.2 | 9 | 11.7 | 12.9 |  |  |  |  |  |  |  |  |
| 260 | 262 | Tro_fonti | 287 | Forat de les Set Cambres | Hortoneda de la Conca | Spain-Lleida | 1.10490 | 42.25423 | 1392 | 7.6 | 2.8 | 7.7 | 10.1 | 11.4 |  |  |  |  |  |  |  |  |
| 261 | 263 | Spn_antemi | 182 | Cova dels Porredons de Baén | Gerri de la Sal | Spain-Lleida | 1.10282 | 42.33086 | 1148 | 8.8 | 4 | 8.9 | 11.3 | 12.6 | MNCN-AI1074 | 2006 | J. Fresneda | GU356891 | GU356789 | HG915615 | GU356935 | GU356982 |
| 262 | 264 | Par_orestes | 474 | grotte de Ramondeuch (= grotte des Poteries) | Seix | France-Ariège | 1.10110 | 42.82677 |  | 5.1 | 0.1 | 5.2 | 7.7 | 9 |  |  |  |  |  |  |  |  |
| 263 | 265 | Spn_tincatincensis | 35 | Forat del Tincatinc | Altrón | Spain-Lleida | 1.10024 | 42.43900 | 1276 | 8 | 3.1 | 8 | 10.5 | 11.8 | NHM-IRC6 | 1999 | J. Fresneda | GU356892 | GU356790 | - | - | GU356983 |
| 264 | 266 | Sty_akarsticus | 35 | Forat del Tincatinc | Altrón | Spain-Lleida | 1.10024 | 42.43900 | 1276 | 8 | 3.1 | 8 | 10.5 | 11.8 | NHM-IRC7 | 1999 | J. Fresneda | GU356899 | GU356798 | GU356845 | GU356940 | GU356990 |
| 265 | 267 | Tro_orcinus | 298 | Avenc del Roc de les Abelles | Farena | Spain-Tarragona | 1.08516 | 41.30883 | 660 | 12.8 | 8.3 | 12.8 | 15.1 | 16.2 |  |  |  |  |  |  |  |  |
| 266 | 268 | Spn_kryophilos | 199 | Forat de les Gralles | Espot | Spain-Lleida | 1.07526 | 42.53349 | 2196 | 2.9 | -2 | 2.9 | 5.4 | 6.7 |  |  |  |  |  |  |  |  |
| 267 | 269 | Tro_fonti | 281 | Forat de la Roqueta | Conca de Dalt | Spain-Lleida | 1.06967 | 42.26999 | 1112 | 9.1 | 4.3 | 9.2 | 11.6 | 12.9 |  |  |  |  |  |  |  |  |
| 268 | 270 | Tro_fonti | 284 | Pou d'Ordins | Abella de la Conca | Spain-Lleida | 1.06778 | 42.17347 | 1412 | 7.5 | 2.8 | 7.6 | 10 | 11.3 |  |  |  |  |  |  |  |  |
| 269 | 271 | Tro_orcinus | 498 | Cova de la Moneda | Montral | Spain-Tarragona | 1.06041 | 41.29296 |  | 11.5 | 7 | 11.5 | 13.8 | 14.9 |  |  |  |  |  |  |  |  |
| 270 | 272 | Tro_orcinus | 300 | Cova Montral | Montral | Spain-Tarragona | 1.05881 | 41.28187 | 1018 | 10.8 | 6.3 | 10.8 | 13.1 | 14.2 |  |  |  |  |  |  |  |  |
| 271 | 273 | Spn_velox | 232 | Cova dels Carrers de Llusàs (=Querant de Picals) | Vilanova de Meià | Spain-Lleida | 1.05781 | 41.99540 |  | 11.8 | 7.1 | 11.9 | 13.8 | 15 |  |  |  |  |  |  |  |  |
| 272 | 274 | Spn_velox | 233 | Querant de Picalts | Vilanova de Meià | Spain-Lleida | 1.05650 | 41.99356 | 766 | 11.3 | 6.6 | 11.4 | 13.8 | 15 |  |  |  |  |  |  |  |  |
| 273 | 275 | Spn_torresi | 196 | Mines de Peramea | Gerri de la Sal | Spain-Lleida | 1.04876 | 42.33245 | 859 | 10.4 | 5.6 | 10.4 | 12.9 | 14.1 |  |  |  |  |  |  |  |  |
| 274 | 276 | Sty_puncticollis | 87 | Avenc del Cingle | Vilanova de Meià | Spain-Lleida | 1.03840 | 42.01867 | 1036 | 9.8 | 5.1 | 9.8 | 12.2 | 13.5 |  |  |  |  |  |  |  |  |
| 275 | 277 | Tro_quadricollis | 87 | Avenc del Cingle | Vilanova de Meià | Spain-Lleida | 1.03840 | 42.01867 | 1036 | 9.8 | 5.1 | 9.8 | 12.2 | 13.5 |  |  |  |  |  |  |  |  |
| 276 | 278 | Tro_vinyasi | 304 | Pou del Dring | Peramola | Spain-Lleida | 1.03840 | 42.01867 | 1036 | 9.8 | 5.1 | 9.8 | 12.2 | 13.5 |  |  |  |  |  |  |  |  |
| 277 | 279 | Sty_puncticollis | 86 | Querant del Pas Nou | Vilanova de Meià | Spain-Lleida | 1.03449 | 42.02700 | 1117 | 9.4 | 4.6 | 9.4 | 11.8 | 13 |  |  |  |  |  |  |  |  |
| 278 | 280 | Spn_velox | 235 | Querant del Riu Merlé (= Cova del riu) | Vilanova de Meià | Spain-Lleida | 1.02888 | 42.00328 | 725 | 11.5 | 6.8 | 11.6 | 14 | 15.2 | MNCN-AI586 | 2002 | J. Fresneda | **LN849275** | **LN849370** | **LN849314** | - | **LN849349** |
| 279 | 281 | Tro_orcinus | 323 | Cova Gran de la Febrò | La Mussara | Spain-Tarragona | 1.02826 | 41.25208 | 969 | 11.1 | 6.6 | 11.1 | 13.4 | 14.5 | NHM-IRC42 | 2002 | F. Fadrique | HF912502 | HF912618/HF912552 | HF912515 | - | HF912600 |
| 280 | 281 | Tro_orcinus | 323 | Cova Gran de la Febrò | La Mussara | Spain-Tarragona | 1.02826 | 41.25208 | 969 | 11.1 | 6.6 | 11.1 | 13.4 | 14.5 | IBE-VR9 | 2009 | V. Rizzo | HF912495 | HF912642/HF912575 | HF912540 | HF912590 | - |
| 281 | 282 | Tro_senenti | 325 | Querant Gran de Paús | Vilanova de Meià | Spain-Barcelona | 1.02258 | 42.01833 | 1090 | 9.5 | 4.8 | 9.6 | 11.9 | 13.2 | MNCN-AI585 | 2002 | J. Fresneda | HF912498 | HF912614/HF912549 | HF912511 | - | HF912596 |
| 282 | 282 | Tro_senenti | 325 | Querant Gran de Paús | Vilanova de Meià | Spain-Barcelona | 1.02258 | 42.01833 | 1090 | 9.5 | 4.8 | 9.6 | 11.9 | 13.2 | IBE-VR6 | 2002 | J. Fresneda | HF912493 | HF912640/HF912573 | HF912538 | - | HF912603 |
| 283 | 283 | Spe_carrerei | 26 | Grotte de Pétillac | Les-Bordes-sur-Lez | France-Ariège | 1.02070 | 42.89557 | 847 | 9.7 | 4.7 | 9.8 | 12.3 | 13.6 | NHM-RB3 |  | Ph. Déliot & A. Faille | **LN849295** | - | **LN849328** | - | - |
| 284 | 284 | Tro_orcinus | 483 | Avenc GIEM | La Febró | Spain-Tarragona | 1.01682 | 41.27010 |  | 11.3 | 6.8 | 11.4 | 13.6 | 14.7 |  |  |  |  |  |  |  |  |
| 285 | 285 | Par_carrerei | 244 | Aven du Trapech d'en Haut (= Puts de l'Hort) | Bordes-sur-Lez | France-Ariège | 1.00754 | 42.82676 | 1600 | 4.8 | -0.2 | 4.9 | 7.4 | 8.7 | IBE-AF184 | 2003 | J. Fresneda | HF912460 | - | HF912507 | HF912580 | HF912592 |
| 286 | 286 | Spe_carrerei | 24 | Grotte de Payssa | Salsein | France-Ariège | 1.00389 | 42.90389 | 847 | 9.7 | 4.7 | 9.8 | 12.2 | 13.6 | MNCN-AI527 | 2004 | A. Faille | **LN849270** | **LN849367** | - | - | - |
| 287 | 287 | Sty_puncticollis | 72 | Forat dels Diners | Gavet de la Conca | Spain-Lleida | 1.00099 | 42.03012 | 1280 | 8.4 | 3.7 | 8.5 | 10.9 | 12.1 |  |  |  |  |  |  |  |  |
| 288 | 288 | Tro_hustachei | 72 | Forat dels Diners | Gavet de la Conca | Spain-Lleida | 1.00099 | 42.03012 | 1280 | 8.4 | 3.7 | 8.5 | 10.9 | 12.1 |  |  |  |  |  |  |  |  |
| 289 | 289 | Sty_akarsticus | 34 | Mines dels Cubilars | Torre de Cabdella | Spain-Lleida | 0.99466 | 42.40578 |  | 7.5 | 2.6 | 7.5 | 10 | 11.3 |  |  |  |  |  |  |  |  |
| 290 | 290 | Tro_fonti | 288 | Cova de les Aranyes | Peracals | Spain-Lleida | 0.98173 | 42.31619 | 1360 | 7.6 | 2.8 | 7.7 | 10.1 | 11.4 |  |  |  |  |  |  |  |  |
| 291 | 291 | Sty_puncticollis | 68 | Baborell de la Dona Morta | Alòs de Balaguer | Spain-Lleida | 0.97825 | 41.93355 | 656 | 12 | 7.3 | 12 | 14.4 | 15.6 |  |  |  |  |  |  |  |  |
| 292 | 292 | Spe_carrerei | 25 | Ravin de la Tire. MSS | Illartein | France-Ariège | 0.97306 | 42.90639 | 1295 | 7.4 | 2.3 | 7.7 | 9.9 | 11.2 | MNCN-AI530 | 2004 | Ph. Déliot & A. Faille | GU356895 | GU356793 | GU356840 | GU356937 | GU356985 |
| 293 | 293 | Sty_akarsticus | 36 | Avenc del Solanet | Astell | Spain-Lleida | 0.97254 | 42.40682 | 1057 | 9.1 | 4.3 | 9.2 | 11.7 | 12.9 |  |  |  |  |  |  |  |  |
| 294 | 294 | Spn_aurouxi | 184 | Cova de Sant Gervàs | Sant Miquel de la Vall | Spain-Lleida | 0.96924 | 42.07173 | 994 | 9.9 | 5.2 | 10 | 12.4 | 13.6 | IBE-AC170 | 2010 | J. Fresneda | - | - | **LN849307** | **LN849336** | **LN849346** |
| 295 | 295 | Spn_aurouxi | 183 | Escletxa Minguera | Sant Miquel de la Vall | Spain-Lleida | 0.96139 | 42.07361 |  | 10.5 | 5.8 | 10.6 | 13 | 14.2 |  |  |  |  |  |  |  |  |
| 296 | 296 | Tro_hustachei | 74 | Forat de la Grallera | Montsec de Rubies | Spain-Lleida | 0.95655 | 42.03661 | 1139 | 9.1 | 4.4 | 9.2 | 11.6 | 12.8 |  |  |  |  |  |  |  |  |
| 297 | 297 | Sty_puncticollis | 74 | Forat de la grallera | Montsec de Rubies | Spain-Lleida | 0.95655 | 42.03661 | 1139 | 9.1 | 4.4 | 9.2 | 11.6 | 12.8 |  |  |  |  |  |  |  |  |
| 298 | 298 | Tro_hustachei | 75 | Cova del Gel | Tremp | Spain-Lleida | 0.95272 | 42.03156 | 1441 | 7.5 | 2.8 | 7.6 | 10 | 11.2 | IBE-VR36 | 2010 | A. Meseguer | HF912487 | HF912635/HF912568 | HF912533 | - | - |
| 299 | 299 | Sty_puncticollis | 75 | Cova del Gel | Tremp | Spain-Lleida | 0.95272 | 42.03156 | 1441 | 7.5 | 2.8 | 7.6 | 10 | 11.2 |  |  |  |  |  |  |  |  |
| 300 | 300 | Spn_crypticola | 188 | Avenc dels Mollons de Lluçà | Senterada | Spain-Lleida | 0.95159 | 42.30967 | 1091 | 9.1 | 4.3 | 9.1 | 11.5 | 12.8 |  |  |  |  |  |  |  |  |
| 301 | 301 | Sty_puncticollis | 67 | Avenc de la Mina | Alòs de Balaguer | Spain-Lleida | 0.95059 | 41.95613 |  | 9.9 | 5.2 | 10 | 12.3 | 13.6 |  |  |  |  |  |  |  |  |
| 302 | 302 | Spn_crypticola | 185 | Cova de Llenes | Erinyà | Spain-Lleida | 0.93278 | 42.31583 |  | 10 | 5.2 | 10.1 | 12.5 | 13.8 |  |  |  |  |  |  |  |  |
| 303 | 303 | Spn_crypticola | 189 | Cova de l'Espluguell | Serradell | Spain-Lleida | 0.90722 | 42.27917 |  | 9 | 4.3 | 9.1 | 11.5 | 12.8 |  |  |  |  |  |  |  |  |
| 304 | 304 | Spn_crypticola | 191 | Forat Negre | Serradell | Spain-Lleida | 0.90560 | 42.27908 | 1092 | 9 | 4.3 | 9.1 | 11.5 | 12.8 | IBE-RA27 | 2009 | J. Fresneda | HG915394 | HG915696 | HG915616 | HG915468 | HG915544 |
| 305 | 305 | Spn_crypticola | 195 | Cova de Toralla | Toralla | Spain-Lleida | 0.90028 | 42.26306 |  | 9 | 4.3 | 9.1 | 11.5 | 12.8 |  |  |  |  |  |  |  |  |
| 306 | 306 | Spn_crypticola | 190 | Forat la Bou | Serradell | Spain-Lleida | 0.89889 | 42.27583 |  | 7.7 | 2.9 | 7.8 | 10.2 | 11.5 |  |  |  |  |  |  |  |  |
| 307 | 307 | Spn_crypticola | 193 | Avenc Barbuixell | Serradell | Spain-Lleida | 0.89406 | 42.27582 | 1340 | 7.7 | 2.9 | 7.8 | 10.2 | 11.5 |  |  |  |  |  |  |  |  |
| 308 | 308 | Spn_nitens | 228 | Avenc de la Figuera | Camarasa | Spain-Lleida | 0.89297 | 41.88997 | 692 | 11.8 | 7.1 | 11.9 | 14.2 | 15.5 |  |  |  |  |  |  |  |  |
| 309 | 309 | Spn_nitens | 229 | Avenc de la Presa | Camarasa | Spain-Lleida | 0.88589 | 41.90642 | 421 | 13.3 | 8.6 | 13.4 | 15.7 | 17 |  |  |  |  |  |  |  |  |
| 310 | 310 | Spn_crypticola | 192 | Graller de Potestats | Serradell | Spain-Lleida | 0.88578 | 42.28170 | 1289 | 7.9 | 3.2 | 8 | 10.5 | 11.7 |  |  |  |  |  |  |  |  |
| 311 | 311 | Sty_puncticollis | 73 | Forat de l'Or | Llimiana | Spain-Lleida | 0.88502 | 42.03901 | 546 | 12.4 | 7.7 | 12.5 | 14.9 | 16.1 | IBE-RA932 | 2012 | J. Fresneda & I. Ribera | **LN849294** | - | **LN849326** | - | **LN849359** |
| 312 | 312 | Spn_nitens | 230 | Cova de l'Escaleta | Camarasa | Spain-Lleida | 0.88345 | 41.90163 | 421 | 13.3 | 8.6 | 13.4 | 15.7 | 17 |  |  |  |  |  |  |  |  |
| 313 | 313 | Sty_puncticollis | 70 | Forat de l'Obaga Massana | Camarasa | Spain-Lleida | 0.88000 | 41.92250 |  | 12.5 | 7.8 | 12.6 | 15 | 16.2 |  |  |  |  |  |  |  |  |
| 314 | 314 | Spn_nitens | 227 | Avenc del Tabaco | Camarasa | Spain-Lleida | 0.87884 | 41.90226 | 378 | 13.6 | 8.9 | 13.6 | 16 | 17.2 | NHM-IRC5 | 2001 | J. Fresneda | **LN849287** | **LN849380** | **LN849319** | - | **LN849353** |
| 315 | 315 | Spn_nitens | 226 | Cova del Tabaco | Camarasa | Spain-Lleida | 0.87847 | 41.90181 | 378 | 13.6 | 8.9 | 13.6 | 16 | 17.2 |  |  |  |  |  |  |  |  |
| 316 | 316 | Sty_puncticollis | 71 | Forat de la Cabeçola | Fontllonga | Spain-Lleida | 0.86553 | 41.97139 | 709 | 11.6 | 6.9 | 11.6 | 14 | 15.2 |  |  |  |  |  |  |  |  |
| 317 | 317 | Spn_latrunculus | 200 | Cova del Lladre | Santa Linya | Spain-Lleida | 0.85878 | 41.90273 | 510 | 12.8 | 8.1 | 12.9 | 15.2 | 16.5 | NHM-IRC8 | 1998 | J. Fresneda | **LN849288** | **LN849381** | **LN849320** | - | **LN849354** |
| 318 | 318 | Sty_aldomai | 37 | Graller de les Planelles de Corroncui | El Pont de Suert | Spain-Lleida | 0.85205 | 42.33680 | 1397 | 7.3 | 2.5 | 7.3 | 9.8 | 11.1 |  |  |  |  |  |  |  |  |
| 319 | 319 | Sty_latebricola | 59 | Forat nº 2 de la Llau Fonda | Tremp | Spain-Lleida | 0.84360 | 42.26053 |  | 7.1 | 2.3 | 7.2 | 9.6 | 10.9 |  |  |  |  |  |  |  |  |
| 320 | 320 | Sty_latebricola | 58 | Esplugallorna de Castellet | Tremp | Spain-Lleida | 0.84171 | 42.27184 | 1459 | 7.1 | 2.3 | 7.1 | 9.6 | 10.8 |  |  |  |  |  |  |  |  |
| 321 | 321 | Sty_latebricola | 57 | Graller de Tremolisses | Tremp | Spain-Lleida | 0.84128 | 42.27279 | 1239 | 7.1 | 2.3 | 7.1 | 10.7 | 12 |  |  |  |  |  |  |  |  |
| 322 | 322 | Sty_latebricola | 61 | Graller de Gurp | Tremp | Spain-Lleida | 0.84049 | 42.24630 | 1410 | 7.3 | 2.5 | 7.4 | 9.8 | 11.1 |  |  |  |  |  |  |  |  |
| 323 | 323 | Sty_puncticollis | 82 | Cova de l'Aigua | Santa Maria de Meià | Spain-Lleida | 0.84020 | 41.89670 | 774 | 11.3 | 6.6 | 11.4 | 13.8 | 15 |  |  |  |  |  |  |  |  |
| 324 | 324 | Sty_latebricola | 54 | Graller del Barranc de la Pedregor | Tremp | Spain-Lleida | 0.83275 | 42.25099 |  | 8.2 | 3.4 | 8.2 | 10.7 | 12 |  |  |  |  |  |  |  |  |
| 325 | 325 | Sty_aldomai | 38 | Cova de Viu de Llevata | El Pont de Suert | Spain-Lleida | 0.82770 | 42.36191 | 1257 | 8 | 3.2 | 8.1 | 10.5 | 11.8 |  |  |  |  |  |  |  |  |
| 326 | 326 | Tro_quadricollis | 60 | Graller de Castellet | Tremp | Spain-Lleida | 0.82373 | 42.25320 | 1025 | 7.6 | 2.8 | 7.7 | 10.1 | 11.4 | MNCN-AI578 | 2003 | J. Fresneda | HF912497 | HF912613/HF912548 | HF912510 | - | HF912595 |
| 327 | 327 | Sty_latebricola | 60 | Graller de Castellet | Tremp | Spain-Lleida | 0.82373 | 42.25320 | 1025 | 7.6 | 2.8 | 7.7 | 10.1 | 11.4 |  |  |  |  |  |  |  |  |
| 328 | 328 | Sty_aldomai | 41 | canchal a 1.100 m en Barruera | Vall de Boí | Spain-Lleida | 0.82328 | 42.51391 | 1116 | 8.6 | 3.7 | 8.6 | 11.1 | 12.4 |  |  |  |  |  |  |  |  |
| 329 | 329 | Sty_latebricola | 53 | Graller del Portús | Tremp | Spain-Lleida | 0.82189 | 42.31617 | 1485 | 6.8 | 2 | 6.9 | 9.3 | 10.6 |  |  |  |  |  |  |  |  |
| 330 | 330 | Sty_puncticollis | 81 | Graller Mitjà del Corralot | Sant Esteve de la Sarga | Spain-Lleida | 0.81942 | 42.04332 | 1381 | 7.7 | 3 | 7.8 | 10.2 | 11.5 |  |  |  |  |  |  |  |  |
| 331 | 331 | Sty_puncticollis | 80 | Graller Gran del Corralot | Sant Esteve de la Sarga | Spain-Lleida | 0.81611 | 42.04481 | 1433 | 7.4 | 2.7 | 7.5 | 9.9 | 11.1 |  |  |  |  |  |  |  |  |
| 332 | 332 | Sty_puncticollis | 79 | Graller del Pas del Llop | Sant Esteve de la Sarga | Spain-Lleida | 0.81354 | 42.04277 | 1433 | 7.4 | 2.7 | 7.5 | 9.9 | 11.1 |  |  |  |  |  |  |  |  |
| 333 | 333 | Sty_latebricola | 51 | Cova del Sanat de Sant Gervàs | Tremp | Spain-Lleida | 0.80074 | 42.31846 | 1787 | 5.2 | 0.4 | 5.2 | 7.7 | 9 |  |  |  |  |  |  |  |  |
| 334 | 334 | Sty_saforensis | 263 | mss 051, en el cruce de Montiverri | El Pont de Suert | Spain-Lleida | 0.79160 | 42.38029 |  | 9.2 | 4.3 | 9.2 | 10.5 | 11.8 |  |  |  |  |  |  |  |  |
| 335 | 335 | Sty_saforensis | 262 | mss 050, en el cruce de Montiverri | El Pont de Suert | Spain-Lleida | 0.79145 | 42.38034 |  | 9.2 | 4.3 | 9.2 | 10.5 | 11.8 |  |  |  |  |  |  |  |  |
| 336 | 336 | Sty_latebricola | 55 | Graller de Sant Roc | El Pont de Suert | Spain-Lleida | 0.78848 | 42.33165 | 1572 | 6.3 | 1.5 | 6.4 | 8.8 | 10.1 | MNCN-AI596 | 2004 | J. Fresneda | **LN849279** | **LN849373** | **LN849316** | **LN849338** | - |
| 337 | 337 | Tro_senenti | 302 | Graller de Espills de Sapeira | Tremp | Spain-Lleida | 0.78755 | 42.23340 | 688 | 10.4 | 4.4 | 10.4 | 12 | 13.3 |  |  |  |  |  |  |  |  |
| 338 | 338 | Sty_puncticollis | 69 | Avenc del Barranc de la Conqueta | Les Avellanes | Spain-Lleida | 0.77955 | 41.93250 | 583 | 12.3 | 7.6 | 12.4 | 14.8 | 16 |  |  |  |  |  |  |  |  |
| 339 | 339 | Sty_aldomai | 39 | Forat d'Irgo | El Pont de Suert | Spain-Lleida | 0.77441 | 42.43353 | 1414 | 7 | 2.2 | 7.1 | 9.6 | 10.9 | MNCN-AI1077 | 2006 | J. Fresneda | HG915406 | HG915708 | HG915630 | HG915477 | HG915557 |
| 340 | 340 | Sty_sanctigervasi | 52 | Mines de Canal de Llastarri | Tremp | Spain-Lleida | 0.77215 | 42.32009 | 1326 | 7.6 | 2.8 | 7.7 | 10.2 | 11.4 | MNCN-AI863 | 2006 | J. Fresneda | **LN849282** | **LN849374** | - | - | **LN849351** |
| 341 | 341 | Sty_latebricola | 52 | Mines de Canal de Llastarri | Tremp | Spain-Lleida | 0.77215 | 42.32009 | 1326 | 7.6 | 2.8 | 7.7 | 10.2 | 11.4 | MNCN-AI862 | 2006 | J. Fresneda | **LN849281** | - | - | - | - |
| 342 | 342 | Sty_sanctigervasi | 96 | Avenc petit de Safor de Llastarri | Tremp | Spain-Lleida | 0.77011 | 42.32116 | 1326 | 7.6 | 2.8 | 7.7 | 10.2 | 11.4 |  |  |  |  |  |  |  |  |
| 343 | 343 | Sty_saforensis | 95 | Avenc de Safor de Llastarri | Tremp | Spain-Lleida | 0.77011 | 42.32116 | 1326 | 7.6 | 2.8 | 7.7 | 10.2 | 11.4 |  |  |  |  |  |  |  |  |
| 344 | 344 | Sty_sanctigervasi | 95 | Avenc de Safor de Llastarri | Tremp | Spain-Lleida | 0.77011 | 42.32116 | 1326 | 7.6 | 2.8 | 7.7 | 10.2 | 11.4 |  |  |  |  |  |  |  |  |
| 345 | 345 | Sty_saforensis | 96 | Avenc petit de Safor de Llastarri | Tremp | Spain-Lleida | 0.77011 | 42.32116 | 1326 | 7.6 | 2.8 | 7.7 | 10.2 | 11.4 |  |  |  |  |  |  |  |  |
| 346 | 346 | Sty_sanctigervasi | 94 | Forat del Pla de Món de Ventolá | El Pont de Suert | Spain-Lleida | 0.76649 | 42.40768 | 1160 | 8.1 | 3.3 | 8.2 | 11 | 12.3 | MNCN-AI1076 | 2006 | J. Fresneda | **LN849283** | - | - | - | - |
| 347 | 347 | Sty_saforensis | 94 | Forat del Pla de Món de Ventolá | El Pont de Suert | Spain-Lleida | 0.76649 | 42.40768 | 1160 | 8.1 | 3.3 | 8.2 | 11 | 12.3 |  |  |  |  |  |  |  |  |
| 348 | 348 | Sty_sanctigervasi | 100 | Forat de l'Aubac de Malpàs | El Pont de Suert | Spain-Lleida | 0.76583 | 42.40590 | 1160 | 8.4 | 3.6 | 8.5 | 11 | 12.3 |  |  |  |  |  |  |  |  |
| 349 | 349 | Sty_puncticollis | 64 | Avenc de Fontdepou | Ager | Spain-Lleida | 0.75741 | 41.95841 | 813 | 10.9 | 6.2 | 11 | 13.4 | 14.6 |  |  |  |  |  |  |  |  |
| 350 | 350 | Sty_puncticollis | 65 | Cova de Fontdepou | Ager | Spain-Lleida | 0.75741 | 41.95841 | 813 | 10.9 | 6.2 | 11 | 13.4 | 14.6 |  |  |  |  |  |  |  |  |
| 351 | 351 | Sty_saforensis | 93 | Forat del camí de Montiverri | El Pont de Suert | Spain-Lleida | 0.75091 | 42.38635 | 946 | 9.6 | 4.8 | 9.7 | 12.2 | 13.5 |  |  |  |  |  |  |  |  |
| 352 | 352 | Sty_sanctigervasi | 93 | Forat del camí de Montiverri | El Pont de Suert | Spain-Lleida | 0.75091 | 42.38635 | 946 | 9.6 | 4.8 | 9.7 | 12.2 | 13.5 |  |  |  |  |  |  |  |  |
| 353 | 353 | Sty_puncticollis | 77 | Graller del Boixaguer | Sant Esteve de la Sarga | Spain-Lleida | 0.74797 | 42.05598 | 1423 | 7.4 | 2.7 | 7.5 | 9.9 | 11.2 |  |  |  |  |  |  |  |  |
| 354 | 354 | Sty_sanctigervasi | 99 | Cova de la Represa | Sopeira | Spain-Huesca | 0.74384 | 42.32437 | 762 | 10.7 | 5.9 | 10.8 | 13.2 | 14.5 |  |  |  |  |  |  |  |  |
| 355 | 355 | Sty_hansferyi | 48 | mss 014, CN 230, PK 117 | Santorens | Spain-Huesca | 0.74311 | 42.35736 | 923 | 9.8 | 4.9 | 9.8 | 12.3 | 13.6 |  |  |  |  |  |  |  |  |
| 356 | 356 | Sty_saforensis | 49 | mss 018, CN 230, PK 117 | Santorens | Spain-Huesca | 0.74311 | 42.35736 | 923 | 9.8 | 4.9 | 9.8 | 12.3 | 13.6 |  |  |  |  |  |  |  |  |
| 357 | 357 | Sty_hansferyi | 49 | mss 018, CN 230, PK 117 | Santorens | Spain-Huesca | 0.74311 | 42.35736 | 923 | 9.8 | 4.9 | 9.8 | 12.3 | 13.6 |  |  |  |  |  |  |  |  |
| 358 | 358 | Sty_saforensis | 48 | mss 014, CN 230, PK 117 | Santorens | Spain-Huesca | 0.74311 | 42.35736 | 923 | 9.8 | 4.9 | 9.8 | 12.3 | 13.6 |  |  |  |  |  |  |  |  |
| 359 | 359 | Sty_sanctigervasi | 98 | Forat de la Cuneta | Sopeira | Spain-Huesca | 0.74252 | 42.32099 | 762 | 10.7 | 5.9 | 10.8 | 13.2 | 14.5 |  |  |  |  |  |  |  |  |
| 360 | 360 | Sty_saforensis | 50 | Cova de la Carretera | El Pont de Suert | Spain-Lleida | 0.74239 | 42.38203 | 828 | 10.3 | 5.4 | 10.3 | 12.8 | 14.1 |  |  |  |  |  |  |  |  |
| 361 | 361 | Sty_hansferyi | 50 | Cova de la Carretera | El Pont de Suert | Spain-Lleida | 0.74239 | 42.38203 | 828 | 10.3 | 5.4 | 10.3 | 12.8 | 14.1 | NHM-IRC39 | 2002 | J. Fresneda | GU356900 | GU356799 | GU356846 | - | GU356991 |
| 362 | 362 | Sty_puncticollis | 78 | Avenc de la Pedró | Sant Esteve de la Sarga | Spain-Lleida | 0.74056 | 42.05278 |  | 7.3 | 2.5 | 7.3 | 9.7 | 11 |  |  |  |  |  |  |  |  |
| 363 | 363 | Sty_sanctigervasi | 264 | mss 052, encima de la presa de Escales | Sopeira | Spain-Huesca | 0.73895 | 42.32781 |  | 9.9 | 5.1 | 10 | 12.4 | 13.7 |  |  |  |  |  |  |  |  |
| 364 | 364 | Sty_saforensis | 92 | Forat de la pista de Buira | Bonansa | Spain-Huesca | 0.73339 | 42.39700 | 963 | 9.5 | 4.7 | 9.6 | 12 | 13.3 |  |  |  |  |  |  |  |  |
| 365 | 365 | Sty_sanctigervasi | 92 | Forat de la pista de Buira | Bonansa | Spain-Huesca | 0.73339 | 42.39700 | 963 | 9.5 | 4.7 | 9.6 | 12 | 13.3 |  |  |  |  |  |  |  |  |
| 366 | 366 | Sty_hansferyi | 46 | Graller Gran del Sodo | Santorens | Spain-Huesca | 0.73108 | 42.34978 | 1054 | 8 | 3.2 | 8.1 | 11.6 | 12.9 |  |  |  |  |  |  |  |  |
| 367 | 367 | Lag_colominasi | 110 | Forat d'Os | Os de Balaguer | Spain-Lleida | 0.72942 | 41.88234 | 553 | 12.5 | 7.8 | 12.6 | 15 | 16.2 |  |  |  |  |  |  |  |  |
| 368 | 368 | Sty_saforensis | 91 | Cova del Garrabero | Bonansa | Spain-Huesca | 0.72894 | 42.37714 | 1251 | 8 | 3.1 | 8 | 10.5 | 11.8 | IBE-AC105 | 2009 | J. Fresneda | **LN849258** | - | **LN849300** | - | - |
| 369 | 369 | Sty_hansferyi | 47 | Pouets de la Canal | Santorens | Spain-Huesca | 0.72858 | 42.35032 | 1247 | 8 | 3.2 | 8.1 | 10.5 | 11.8 |  |  |  |  |  |  |  |  |
| 370 | 370 | Sty_zariquieyi | 105 | Graller de Badià | Ager | Spain-Lleida | 0.72521 | 42.04108 | 1170 | 8.8 | 4.1 | 8.9 | 11.3 | 12.6 |  |  |  |  |  |  |  |  |
| 371 | 371 | Sty_aldomai | 40 | Forat de Sarroqueta | El Pont de Suert | Spain-Lleida | 0.72506 | 42.44181 | 1191 | 8.2 | 3.3 | 8.3 | 10.7 | 12.1 |  |  |  |  |  |  |  |  |
| 372 | 372 | Lag_colominasi | 265 | Cova del Foric | Os de Balaguer | Spain-Lleida | 0.71810 | 41.87460 |  | 13.1 | 8.4 | 13.2 | 15.6 | 16.8 |  |  |  |  |  |  |  |  |
| 373 | 373 | Sty_puncticollis | 76 | Graller de Corona | Sant Esteve de la Sarga | Spain-Lleida | 0.71807 | 42.05364 | 1493 | 7 | 2.3 | 7.1 | 9.5 | 10.8 |  |  |  |  |  |  |  |  |
| 374 | 374 | Lag_colominasi | 106 | Avenc de la Penya dels Dos Ulls | Os de Balaguer | Spain-Lleida | 0.71388 | 41.86832 | 505 | 12.8 | 8.1 | 12.9 | 15.3 | 16.5 |  |  |  |  |  |  |  |  |
| 375 | 375 | Sty_hansferyi | 45 | Esplugafonda | Betesa | Spain-Huesca | 0.69303 | 42.35920 | 1150 | 8.5 | 3.7 | 8.6 | 11 | 12.3 | MNCN-AI593 | 2004 | J. Fresneda | **LN849278** | **LN849372** | - | - | - |
| 376 | 376 | Sty_ribagorzanus | 89 | Cova des Toscllasses = Cova de les Tollasses. Cova des Cllosses | Bonansa | Spain-Huesca | 0.69173 | 42.42432 | 1197 | 8.2 | 3.3 | 8.2 | 10.7 | 12 | MNCN-AI575 | 2004 | J. Fresneda | **LN849272** | **LN849368** | **LN849313** | - | **LN849348** |
| 377 | 377 | Lag_colominasi | 108 | Forat del Patxarro | Os de Balaguer | Spain-Lleida | 0.68569 | 41.91872 | 699 | 11.6 | 6.9 | 11.7 | 14.1 | 15.3 |  |  |  |  |  |  |  |  |
| 378 | 378 | Sty_espinosai | 42 | Coves de Berganui | Areny de Noguera | Spain-Huesca | 0.68411 | 42.26100 | 1046 | 9.2 | 4.4 | 9.2 | 11.7 | 13 |  |  |  |  |  |  |  |  |
| 379 | 379 | Sty_zariquieyi | 103 | Cova Negra de Corsà | Ager | Spain-Lleida | 0.68322 | 42.06865 | 729 | 8.9 | 4.1 | 8.9 | 13.7 | 14.9 |  |  |  |  |  |  |  |  |
| 380 | 380 | Sty_zariquieyi | 104 | Cova Colomera de Corsà | Ager | Spain-Lleida | 0.68176 | 42.07880 | 578 | 12 | 7.3 | 12.1 | 14.5 | 15.8 | IBE-AF187 | 2009 | J. Fresneda | **LN849268** | **LN849364** | **LN849311** | **LN849337** | **LN849347** |
| 381 | 381 | Lag_colominasi | 107 | Cova Joan d'Os (= Cova d’Ullet) | Os de Balaguer | Spain-Lleida | 0.67764 | 41.92322 | 840 | 10.8 | 6.1 | 10.8 | 13.3 | 14.5 | NHM-IRC30 | 2001 | J. Fresneda | GU356869 | GU356765 | GU356819 | GU356918 | GU356961 |
| 382 | 382 | Sty_hansferyi | 43 | Cova de Casa Pallàs | Betesa | Spain-Huesca | 0.67152 | 42.34225 | 1350 | 7.4 | 2.6 | 7.5 | 9.9 | 11.2 | MNCN-AI599 | 2004 | J. Fresneda | **LN849280** | - | - | - | - |
| 383 | 383 | Sty_hansferyi | 44 | Cova d'Obís | Betesa | Spain-Huesca | 0.66824 | 42.35880 | 1403 | 7.1 | 2.3 | 7.2 | 9.7 | 11 |  |  |  |  |  |  |  |  |
| 384 | 384 | Tra_articollis | 88 | Cova de Sant Salvador de Bibils | Bonansa | Spain-Huesca | 0.65303 | 42.43743 | 1427 | 6.9 | 2.1 | 7 | 9.5 | 10.8 | MNCN-AI576 | 2003 | J. Fresneda | **LN849273** | **LN849369** | - | - | - |
| 385 | 385 | Sty_ribagorzanus | 88 | Cova de Sant Salvador de Bibils | Bonansa | Spain-Huesca | 0.65303 | 42.43743 | 1427 | 6.9 | 2.1 | 7 | 9.5 | 10.8 | MNCN-AI577 | 2003 | J. Fresneda | **LN849274** | - | - | - | - |
| 386 | 386 | Tra_cerberus | 165 | Esplluga de les Tosses | Bonansa | Spain-Huesca | 0.64909 | 42.41231 | 1730 | 5.3 | 0.4 | 5.3 | 7.8 | 9.1 | MNCN-AI600 | 2004 | J. Fresneda | HF912499 | HF912615/HF912550 | HF912512 | HF912583 | HF912597 |
| 387 | 387 | Sty_puncticollis | 66 | Forat la Neu en la Serra de Blancafort | Ager | Spain-Lleida | 0.64451 | 41.96418 | 804 | 10.9 | 6.2 | 11 | 13.4 | 14.6 |  |  |  |  |  |  |  |  |
| 388 | 388 | Sty_ribagorzanus | 90 | Espluga dels Feixants | Bonansa | Spain-Huesca | 0.64146 | 42.42183 | 1488 | 6.9 | 2.1 | 7 | 9.1 | 10.4 |  |  |  |  |  |  |  |  |
| 389 | 389 | Tra_cerberus | 90 | Espluga dels Feixants | Bonansa | Spain-Huesca | 0.64146 | 42.42183 | 1488 | 6.9 | 2.1 | 7 | 9.1 | 10.4 |  |  |  |  |  |  |  |  |
| 390 | 390 | Lag_colominasi | 111 | Avenc de la Plana | Tragó de Noguera | Spain-Lleida | 0.62644 | 41.90418 | 626 | 12 | 7.3 | 12.1 | 14.5 | 15.7 |  |  |  |  |  |  |  |  |
| 391 | 391 | Sty_puncticollis | 85 | Cova Fonda de Tragó | Tragó de Noguera | Spain-Lleida | 0.62426 | 41.94803 | 638 | 11.9 | 7.2 | 11.9 | 14.3 | 15.6 |  |  |  |  |  |  |  |  |
| 392 | 392 | Sty_puncticollis | 84 | Cova Negra de Tragó | Tragó de Noguera | Spain-Lleida | 0.61402 | 41.97580 | 494 | 12.6 | 7.9 | 12.7 | 15.1 | 16.3 |  |  |  |  |  |  |  |  |
| 393 | 393 | Tra_cerberus | 166 | mss 065, en Espés de Baix | Laspaúles | Spain-Huesca | 0.60678 | 42.43189 | 1407 | 7 | 2.1 | 7 | 9.5 | 10.8 |  |  |  |  |  |  |  |  |
| 394 | 394 | Tra_orobios | 174 | Clot de la Bassa de Gabás | Bisaurri | Spain-Huesca | 0.49702 | 42.46000 | 1561 | 6.1 | 1.2 | 6.1 | 8.6 | 9.9 | MNCN-AI589 | 2004 | J. Fresneda | **LN849276** | **LN849371** | **LN849315** | - | **LN849350** |
| 395 | 395 | Tra_orobios | 178 | Sumidero en una riera cerca del Collau Plana del Turbón. 1.920 m. | Egea | Spain-Huesca | 0.49376 | 42.41150 | 2000 | 3.8 | -1.1 | 3.8 | 6.3 | 7.6 |  |  |  |  |  |  |  |  |
| 396 | 396 | Tra_gimenezi | 169 | Forau de las Grallas del Turbón (= El Grallero) | Egea | Spain-Huesca | 0.49058 | 42.41163 | 1976 | 3.9 | -1 | 3.9 | 6.4 | 7.7 |  |  |  |  |  |  |  |  |
| 397 | 397 | Tra_orobios | 177 | Sumidero en el Collau de la Plana del Turbón. 2.000 m. | Egea | Spain-Huesca | 0.48896 | 42.41610 | 1976 | 3.9 | -1 | 3.9 | 6.4 | 7.7 | MNCN-AI591 | 2004 | J. Fresneda | **LN849277** | - | - | - | - |
| 398 | 398 | Tra_carrodillae | 162 | Forat de l'Aire | Camporrells | Spain-Huesca | 0.47090 | 41.99415 | 986 | 9.7 | 5 | 9.8 | 12.2 | 13.5 |  |  |  |  |  |  |  |  |
| 399 | 399 | Lag_porroinensis | 114 | Sia del Tossal de Felis | Purroi de la Solana | Spain-Huesca | 0.46201 | 42.05186 | 732 | 11 | 6.3 | 11.1 | 13.5 | 14.8 |  |  |  |  |  |  |  |  |
| 400 | 400 | Tra_carrodillae | 163 | Forat de les Guitarres | Camporrells | Spain-Huesca | 0.45019 | 42.51780 | 1379 | 6.9 | 2 | 7 | 9.5 | 10.8 |  |  |  |  |  |  |  |  |
| 401 | 401 | Tra_orobios | 180 | Sierra de Chía, dolina-sumidero de Risuali | Chía | Spain-Huesca | 0.45019 | 42.51780 | 1379 | 6.9 | 2 | 7 | 9.5 | 10.8 |  |  |  |  |  |  |  |  |
| 402 | 402 | Tra_orobios | 181 | mss 042 | Chía | Spain-Huesca | 0.44373 | 42.53102 | 1557 | 6 | 1.1 | 6.1 | 8.5 | 9.9 |  |  |  |  |  |  |  |  |
| 403 | 403 | Tra_orobios | 175 | Cervín. mss 053 | Campo | Spain-Huesca | 0.43114 | 42.44030 | 1469 | 6.5 | 1.7 | 6.6 | 9.1 | 10.4 | IBE-AF55 | 2008 | A. Faille & J. Fresneda | **LN849264** | - | - | - | - |
| 404 | 404 | Tra_orobios | 176 | Cervín. mss 054 | Campo | Spain-Huesca | 0.42924 | 42.44110 | 1469 | 6.5 | 1.7 | 6.6 | 9.1 | 10.4 |  |  |  |  |  |  |  |  |
| 405 | 405 | Tra_escollae | 167 | mss 004. 750 m., CN 260 pk: 396-397 | Seira | Spain-Huesca | 0.40442 | 42.46346 | 880 | 9.6 | 4.7 | 9.7 | 12.1 | 13.5 |  |  |  |  |  |  |  |  |
| 406 | 406 | Tra_escollae | 168 | mss 037, CN 260 pk: 400 | Viu | Spain-Huesca | 0.40207 | 42.46110 | 880 | 9.6 | 4.7 | 9.7 | 12.1 | 13.5 |  |  |  |  |  |  |  |  |
| 407 | 407 | Tra_bolivari | 149 | mss 002, CN 260, PK 396.5, 750 m. | Seira | Spain-Huesca | 0.40193 | 42.46114 | 880 | 9.6 | 4.7 | 9.7 | 12.1 | 13.5 | IBE-AF128 | 2009 | J. Fresneda, A. Faille & C. Bourdeau | - | **LN849362** | - | - | - |
| 408 | 408 | Tra_escollae | 149 | mss 002, CN 260, PK 396.5 | Seira | Spain-Huesca | 0.40193 | 42.46114 | 880 | 9.6 | 4.7 | 9.7 | 12.1 | 13.5 | IBE-AF129 | 2009 | J. Fresneda, A. Faille & C. Bourdeau | **LN849266** | **LN849363** | **LN849310** | - | - |
| 409 | 409 | Tra_bolivari | 126 | Cueva de la pista del Caixigar. cerca de Punta Naspún | Campo | Spain-Huesca | 0.38627 | 42.39940 | 954 | 9.3 | 4.5 | 9.4 | 11.8 | 13.2 |  |  |  |  |  |  |  |  |
| 410 | 410 | Nas_eseranus | 115 | Inflas de Naspún (= Avenc del Toscar) | Foradada del Toscar | Spain-Huesca | 0.37849 | 42.40063 | 1017 | 8.9 | 4.1 | 9 | 11.5 | 12.8 | NHM-IRC29 | 2001 | J. Fresneda | GU356872 | GU356768 | GU356821 | GU356920 | GU356963 |
| 411 | 411 | Tra_bolivari | 115 | Inflas de Naspún (= Avenc del Toscar) | Foradada del Toscar | Spain-Huesca | 0.37849 | 42.40063 | 1017 | 8.9 | 4.1 | 9 | 11.5 | 12.8 | NHM-IRC25 | 2001 | J. Fresneda | **LN849289** | **LN849377** | **LN849321** | - | - |
| 412 | 412 | Tra_bolivari | 161 | mss 045, entre Senz y Viu | Viu | Spain-Huesca | 0.34984 | 42.43995 | 1170 | 9 | 4.1 | 9.1 | 10.6 | 12 |  |  |  |  |  |  |  |  |
| 413 | 413 | Tra_bolivari | 160 | mss 044. entre Senz y Viu | Viu | Spain-Huesca | 0.34984 | 42.43995 | 1170 | 9 | 4.1 | 9.1 | 10.6 | 12 |  |  |  |  |  |  |  |  |
| 414 | 414 | Tra_orobios | 173 | Circo de Armeña, Sima A-182 | Barbaruens | Spain-Huesca | 0.34402 | 42.51166 | 2177 | 2.6 | -2.3 | 2.7 | 5.2 | 6.5 |  |  |  |  |  |  |  |  |
| 415 | 415 | Tra_orobios | 172 | Circo de Armeña, sima A-332 | Barbaruens | Spain-Huesca | 0.33991 | 42.51747 | 2155 | 2.8 | -2.2 | 2.8 | 5.3 | 6.7 | IBE-AF59 | 2008 | J. Fresneda, A. Faille & C. Bourdeau | HG915407 | HG915709 | HG915631 | HG915478 | HG915558 |
| 416 | 416 | Tra_bolivari | 140 | mss 005, CN 260 PK 413,5, Collau de Foradada | Foradada del Toscar | Spain-Huesca | 0.33960 | 42.41302 | 1017 | 8.9 | 4 | 9 | 11.4 | 12.8 |  |  |  |  |  |  |  |  |
| 417 | 417 | Tra_bolivari | 141 | mss 007, CN 260 PK 414, Collau de Foradada | Foradada del Toscar | Spain-Huesca | 0.33903 | 42.41327 | 1017 | 8.9 | 4 | 9 | 11.4 | 12.8 |  |  |  |  |  |  |  |  |
| 418 | 418 | Tra_bolivari | 157 | Infla de la Estiva | Toledo de la Nata | Spain-Huesca | 0.28056 | 42.46784 | 1765 | 4.9 | 0 | 4.9 | 7.4 | 8.8 |  |  |  |  |  |  |  |  |
| 419 | 419 | Tra_bolivari | 151 | mss 056 | Sin | Spain-Huesca | 0.27634 | 42.58102 | 1161 | 7.9 | 3 | 8 | 10.5 | 11.9 |  |  |  |  |  |  |  |  |
| 420 | 420 | Tra_bolivari | 152 | mss 058 | Sin | Spain-Huesca | 0.26997 | 42.56501 | 988 | 8.8 | 4 | 8.9 | 11.4 | 12.8 |  |  |  |  |  |  |  |  |
| 421 | 421 | Tra_bolivari | 153 | mss 059 | Sin | Spain-Huesca | 0.26997 | 42.56501 | 988 | 8.8 | 4 | 8.9 | 11.4 | 12.8 |  |  |  |  |  |  |  |  |
| 422 | 422 | Tra_carrodillae | 164 | Grallera de Estadilla | Estadilla | Spain-Huesca | 0.26436 | 42.06070 | 606 | 11.5 | 6.8 | 11.6 | 14.1 | 15.4 | NHM-IRC9 | 1999 | J. Fresneda | GU356901 | GU356800 | GU356847 | - | GU356992 |
| 423 | 423 | Tra_orobios | 170 | Cueva Graners | Badaín | Spain-Huesca | 0.23685 | 42.52455 | 1181 | 7.9 | 3 | 7.9 | 10.4 | 11.8 |  |  |  |  |  |  |  |  |
| 424 | 424 | Tra_orobios | 171 | Cueva Pot au Feu | Badaín | Spain-Huesca | 0.23582 | 42.52438 | 1181 | 7.9 | 3 | 7.9 | 10.4 | 11.8 | IBE-AF180 | 2009 | J. Fresneda | **LN849267** | - | - | - | - |
| 425 | 425 | Tra_bolivari | 143 | Cueva del Hueso Santo de Oncins. 1.300 m | El Pueyo de Araguás | Spain-Huesca | 0.21127 | 42.47171 | 1331 | 7.1 | 2.3 | 7.2 | 9.7 | 11.1 | MNCN-HI23 | 2006 | J. Fresneda | **LN849284** | **LN849375** | **LN849317** | - | **LN849352** |
| 426 | 426 | Tra_bolivari | 142 | Cueva de las Devotas | Lafortunada | Spain-Huesca | 0.20466 | 42.55810 | 811 | 9 | 4.1 | 9.1 | 12.3 | 13.7 |  |  |  |  |  |  |  |  |
| 427 | 427 | Tra_bolivari | 132 | Sima B-1 | Escuaín | Spain-Huesca | 0.12634 | 42.60196 | 1359 | 6.8 | 1.9 | 6.9 | 9.4 | 10.8 |  |  |  |  |  |  |  |  |
| 428 | 428 | Tra_orobios | 132 | Sima B-1 | Escuaín | Spain-Huesca | 0.12634 | 42.60196 | 1359 | 6.8 | 1.9 | 6.9 | 9.4 | 10.8 |  |  |  |  |  |  |  |  |
| 429 | 429 | Tra_orobios | 261 | Sima B-15 | Escuaín | Spain-Huesca | 0.12132 | 42.62489 | 2008 | 1.2 | -3.8 | 1.3 | 5.9 | 7.3 | IBE-RA958 | 2012 | A. Faille | - | **LN849366** | **LN849327** | - | **LN849360** |
| 430 | 430 | Tra_orobios | 122 | Sima B-26 | Escuaín | Spain-Huesca | 0.11669 | 42.62078 | 2008 | 3.4 | -1.6 | 3.4 | 5.9 | 7.3 |  |  |  |  |  |  |  |  |
| 431 | 431 | Tra_bolivari | 127 | Avenc B-12 | Escuaín | Spain-Huesca | 0.11358 | 42.62720 | 2186 | 2.4 | -2.5 | 2.5 | 5 | 6.3 |  |  |  |  |  |  |  |  |
| 432 | 432 | Tra_orobios | 123 | Sumidero de Gurrundué | Escuaín | Spain-Huesca | 0.09982 | 42.62711 | 2031 | 3.7 | -1.3 | 3.7 | 5.8 | 7.2 |  |  |  |  |  |  |  |  |
| 433 | 433 | Tra_bolivari | 128 | Avenc de la Bufona | Escuaín | Spain-Huesca | 0.09257 | 42.62543 | 2031 | 3.2 | -1.7 | 3.3 | 5.8 | 7.2 |  |  |  |  |  |  |  |  |
| 434 | 434 | Tra_bolivari | 150 | Cueva de Aso (= Cueva del Molino o Cueva de los Moros) | Sercué | Spain-Huesca | 0.03767 | 42.56377 | 1115 | 8.1 | 3.2 | 8.2 | 10.6 | 12 | NHM-IRC27 | 2001 | J. Fresneda | - | **LN849378** | **LN849322** | **LN849340** | **LN849355** |
| 435 | 435 | Tra_bolivari | 136 | Cueva Superior de las Gloces (= Cueva de la Peña = Cueva de los Moros) | Fanlo | Spain-Huesca | 0.02831 | 42.59420 | 1991 | 3.5 | -1.5 | 3.5 | 6 | 7.4 |  |  |  |  |  |  |  |  |
| 436 | 436 | Tra_bolivari | 135 | Cueva inferior de las Gloces | Fanlo | Spain-Huesca | 0.02831 | 42.59420 | 1991 | 3.5 | -1.5 | 3.5 | 6 | 7.4 |  |  |  |  |  |  |  |  |
| 437 | 437 | Sal_brieti | 119 | Cueva de Don Macario en Burgasé | Ginuábel | Spain-Huesca | 0.01648 | 42.51716 | 1266 | 8.2 | 3.3 | 8.2 | 9.9 | 11.2 |  |  |  |  |  |  |  |  |
| 438 | 438 | Tro_rovirai | 119 | Cueva de Don Macario en Burgasé | Ginuábel | Spain-Huesca | 0.01648 | 42.51716 | 1266 | 8.2 | 3.3 | 8.2 | 9.9 | 11.2 |  |  |  |  |  |  |  |  |
| 439 | 439 | Tra_orobios | 121 | Cueva de Garsés | Torla | Spain-Huesca | 0.01528 | 42.65215 | 2048 | 3.1 | -1.9 | 3.1 | 5.7 | 7 |  |  |  |  |  |  |  |  |
| 440 | 440 | Sal_brieti | 117 | Forato de los Moros | Ginuábel | Spain-Huesca | -0.04735 | 42.47818 | 824 | 9.6 | 4.7 | 9.7 | 12.2 | 13.6 | MNCN-AI587 | 2002 | J. Fresneda | GU356885 | GU356783 | GU356832 | GU356930 | GU356977 |
| 441 | 441 | Sal_brieti | 118 | Cueva de Burgasé | Ginuábel | Spain-Huesca | -0.04735 | 42.47818 | 824 | 9.6 | 4.7 | 9.7 | 12.2 | 13.6 |  |  |  |  |  |  |  |  |
| 442 | 442 | Sal_brieti | 116 | Espluga de Barrau | Ginuábel | Spain-Huesca | -0.04735 | 42.47818 | 824 | 9.6 | 4.7 | 9.7 | 12.2 | 13.6 |  |  |  |  |  |  |  |  |
| 443 | 443 | Tra_bolivari | 158 | Cueva de Bujaruelo | Torla | Spain-Huesca | -0.11742 | 42.66520 | 1485 | 6 | 1.1 | 6.1 | 8.6 | 10 |  |  |  |  |  |  |  |  |
| 445 | 445 | Tro_ferreri | 378 | Avenc Joan Cabeza | Begues | Spain-Barcelona | 1.91719 | 41.31268 | 489 | 14.2 | 9.8 | 14.2 | 16.4 | 17.4 |  |  |  |  |  |  |  |  |
| 444 |  | Tro_ferreri |  | Av. Gran de les Alzines | Vallirana | Spain-Barcelona |  |  |  |  |  |  |  |  | IBE-RA748 | 2012 | J. Pastor | **LN849293** | - | - | - | - |

**Table S3** **Data on past and current temperature for each species.** Average and range of Mean Annual Temperature (MAT) for each species (i.e. monophyletic units), considering both current and Last Glacial Maximum (LGM) conditions. Coded_species, code of the monophyletic units used (see Supplementary Table S1). The number of caves in which each species occurs is also indicated (N. caves).

| No | Coded_species | N.  caves | Average of  MAT  (current) | Max of MAT  (current) | Min of MAT  (current) | Average of MAT  (LGM) | Max of MAT  (LGM) | Min of MAT  (LGM) | Current  Range | Historical  Range | MAT  (current-LGM) |
| --- | --- | --- | --- | --- | --- | --- | --- | --- | --- | --- | --- |
| 1 | Ant_dispar | 5 | 10.84 | 12.20 | 9.00 | 5.84 | 7.2 | 4.10 | 3.20 | 8.10 | 5.00 |
| 2 | Ant_querilhaci | 4 | 9.55 | 12.00 | 6.20 | 4.63 | 7.1 | 1.30 | 5.80 | 10.70 | 4.93 |
| 3 | Cer_cenarroi | 2 | 8.40 | 9.00 | 7.80 | 3.70 | 4.3 | 3.10 | 1.20 | 5.90 | 4.70 |
| 4 | Cer_riberai | 5 | 9.20 | 9.20 | 9.20 | 4.50 | 4.5 | 4.50 | 0.00 | 4.70 | 4.70 |
| 5 | Ges_delioti | 1 | 8.40 | 8.40 | 8.40 | 3.50 | 3.5 | 3.50 | 0.00 | 4.90 | 4.90 |
| 6 | Lag_colominasi | 6 | 12.13 | 13.10 | 10.80 | 7.43 | 8.4 | 6.10 | 2.30 | 7.00 | 4.70 |
| 7 | Lag_porroinensis | 1 | 11.00 | 11.00 | 11.00 | 6.30 | 6.3 | 6.30 | 0.00 | 4.70 | 4.70 |
| 8 | Met_monticola | 2 | 8.80 | 10.90 | 6.70 | 3.90 | 6 | 1.80 | 4.20 | 9.10 | 4.90 |
| 9 | Nas_eseranus | 1 | 8.90 | 8.90 | 8.90 | 4.10 | 4.1 | 4.10 | 0.00 | 4.80 | 4.80 |
| 10 | Pal_pallaresana | 1 | 11.00 | 11.00 | 11.00 | 6.20 | 6.2 | 6.20 | 0.00 | 4.80 | 4.80 |
| 11 | Par_carrerei | 1 | 4.80 | 4.80 | 4.80 | -0.20 | -0.2 | -0.20 | 0.00 | 5.00 | 5.00 |
| 12 | Par_jeanneli | 1 | 6.70 | 6.70 | 6.70 | 1.80 | 1.8 | 1.80 | 0.00 | 4.90 | 4.90 |
| 13 | Par_orestes | 1 | 5.10 | 5.10 | 5.10 | 0.10 | 0.1 | 0.10 | 0.00 | 5.00 | 5.00 |
| 14 | Pas_vandeli | 1 | 10.40 | 10.40 | 10.40 | 5.40 | 5.4 | 5.40 | 0.00 | 5.00 | 5.00 |
| 15 | Per_bofilli | 2 | 5.00 | 6.20 | 3.80 | 0.25 | 1.5 | -1.00 | 2.40 | 7.20 | 4.75 |
| 16 | Per_faurai | 1 | 9.50 | 9.50 | 9.50 | 4.80 | 4.8 | 4.80 | 0.00 | 4.70 | 4.70 |
| 17 | Per_fresnedai | 1 | 0.70 | 0.70 | 0.70 | -4.10 | -4.1 | -4.10 | 0.00 | 4.80 | 4.80 |
| 18 | Sal_brieti | 4 | 9.25 | 9.60 | 8.20 | 4.35 | 4.7 | 3.30 | 1.40 | 6.30 | 4.90 |
| 19 | Spe_abeillei | 2 | 10.40 | 11.70 | 9.10 | 5.40 | 6.6 | 4.20 | 2.60 | 7.50 | 5.00 |
| 20 | Spe_bonvouloiri | 1 | 12.80 | 12.80 | 12.80 | 8.00 | 8 | 8.00 | 0.00 | 4.80 | 4.80 |
| 21 | Spe_carrerei | 3 | 8.93 | 9.70 | 7.40 | 3.90 | 4.7 | 2.30 | 2.30 | 7.40 | 5.03 |
| 22 | Spe_chardonis | 3 | 11.80 | 12.10 | 11.40 | 6.80 | 7.1 | 6.50 | 0.70 | 5.60 | 5.00 |
| 23 | Spe_colluvii | 4 | 8.83 | 10.90 | 7.50 | 3.90 | 6 | 2.60 | 3.40 | 8.30 | 4.93 |
| 24 | Spe_curvipes | 4 | 10.23 | 10.70 | 9.90 | 5.33 | 5.8 | 5.00 | 0.80 | 5.70 | 4.90 |
| 25 | Spe_diecki | 1 | 10.60 | 10.60 | 10.60 | 5.60 | 5.6 | 5.60 | 0.00 | 5.00 | 5.00 |
| 26 | Spe_fagniezi | 3 | 12.13 | 12.90 | 10.60 | 7.27 | 8.1 | 5.60 | 2.30 | 7.30 | 4.87 |
| 27 | Spe_longicornis | 5 | 10.80 | 12.20 | 8.20 | 5.82 | 7.2 | 3.30 | 4.00 | 8.90 | 4.98 |
| 28 | Spe_normandi | 3 | 10.90 | 12.00 | 9.60 | 5.90 | 7 | 4.60 | 2.40 | 7.40 | 5.00 |
| 29 | Spe_piochardi | 2 | 10.80 | 11.50 | 10.10 | 5.85 | 6.5 | 5.20 | 1.40 | 6.30 | 4.95 |
| 30 | Spe_proserpinae | 1 | 10.40 | 10.40 | 10.40 | 5.50 | 5.5 | 5.50 | 0.00 | 4.90 | 4.90 |
| 31 | Spe_pyreneus | 6 | 11.30 | 12.00 | 9.80 | 6.32 | 7.1 | 4.80 | 2.20 | 7.20 | 4.98 |
| 32 | Spe_stygius | 6 | 11.07 | 11.70 | 9.80 | 6.07 | 6.7 | 4.80 | 1.90 | 6.90 | 5.00 |
| 33 | Spe_zophosinus | 2 | 11.10 | 11.10 | 11.10 | 6.20 | 6.2 | 6.20 | 0.00 | 4.90 | 4.90 |
| 34 | Spn_andorranus | 2 | 9.40 | 9.40 | 9.40 | 4.50 | 4.5 | 4.50 | 0.00 | 4.90 | 4.90 |
| 35 | Spn_antemi | 1 | 8.80 | 8.80 | 8.80 | 4.00 | 4 | 4.00 | 0.00 | 4.80 | 4.80 |
| 36 | Spn_aurouxi | 2 | 10.20 | 10.50 | 9.90 | 5.50 | 5.8 | 5.20 | 0.60 | 5.30 | 4.70 |
| 37 | Spn_crypticola | 9 | 8.93 | 11.00 | 7.70 | 4.18 | 6.2 | 2.90 | 3.30 | 8.10 | 4.76 |
| 38 | Spn_kryophilos | 1 | 2.90 | 2.90 | 2.90 | -2.00 | -2 | -2.00 | 0.00 | 4.90 | 4.90 |
| 39 | Spn_latrunculus | 1 | 12.80 | 12.80 | 12.80 | 8.10 | 8.1 | 8.10 | 0.00 | 4.70 | 4.70 |
| 40 | Spn_mengeli | 20 | 9.19 | 12.30 | 6.20 | 4.48 | 7.6 | 1.50 | 6.10 | 10.80 | 4.71 |
| 41 | Spn_nitens | 5 | 13.12 | 13.60 | 11.80 | 8.42 | 8.9 | 7.10 | 1.80 | 6.50 | 4.70 |
| 42 | Spn_tincatincensis | 1 | 8.00 | 8.00 | 8.00 | 3.10 | 3.1 | 3.10 | 0.00 | 4.90 | 4.90 |
| 43 | Spn_torresi | 1 | 10.40 | 10.40 | 10.40 | 5.60 | 5.6 | 5.60 | 0.00 | 4.80 | 4.80 |
| 44 | Spn_velox | 3 | 11.53 | 11.80 | 11.30 | 6.83 | 7.1 | 6.60 | 0.50 | 5.20 | 4.70 |
| 45 | Sty_akarsticus | 3 | 8.20 | 9.10 | 7.50 | 3.33 | 4.3 | 2.60 | 1.60 | 6.50 | 4.87 |
| 46 | Sty_aldomai | 5 | 7.82 | 8.60 | 7.00 | 2.98 | 3.7 | 2.20 | 1.60 | 6.40 | 4.84 |
| 47 | Sty_espinosai | 1 | 9.20 | 9.20 | 9.20 | 4.40 | 4.4 | 4.40 | 0.00 | 4.80 | 4.80 |
| 48 | Sty_hansferyi | 8 | 8.61 | 10.30 | 7.10 | 3.78 | 5.4 | 2.30 | 3.20 | 8.00 | 4.84 |
| 49 | Sty_latebricola | 10 | 7.03 | 8.20 | 5.20 | 2.23 | 3.4 | 0.40 | 3.00 | 7.80 | 4.80 |
| 50 | Sty_puncticollis | 23 | 9.90 | 12.60 | 7.00 | 5.19 | 7.9 | 2.30 | 5.60 | 10.30 | 4.71 |
| 51 | Sty_ribagorzanus | 3 | 7.33 | 8.20 | 6.90 | 2.50 | 3.3 | 2.10 | 1.30 | 6.10 | 4.83 |
| 52 | Sty_saforensis | 11 | 8.97 | 10.30 | 7.60 | 4.12 | 5.4 | 2.80 | 2.70 | 7.50 | 4.85 |
| 53 | Sty_sanctigervasi | 10 | 8.97 | 10.70 | 7.60 | 4.17 | 5.9 | 2.80 | 3.10 | 7.90 | 4.80 |
| 54 | Sty_zariquieyi | 3 | 9.90 | 12.00 | 8.80 | 5.17 | 7.3 | 4.10 | 3.20 | 7.90 | 4.73 |
| 55 | Tra_articollis | 1 | 6.90 | 6.90 | 6.90 | 2.10 | 2.1 | 2.10 | 0.00 | 4.80 | 4.80 |
| 56 | Tra_bolivari | 20 | 7.18 | 9.60 | 2.40 | 2.30 | 4.7 | -2.50 | 7.20 | 12.10 | 4.89 |
| 57 | Tra_carrodillae | 3 | 9.37 | 11.50 | 6.90 | 4.60 | 6.8 | 2.00 | 4.60 | 9.50 | 4.77 |
| 58 | Tra_cerberus | 3 | 6.40 | 7.00 | 5.30 | 1.53 | 2.1 | 0.40 | 1.70 | 6.60 | 4.87 |
| 59 | Tra_escollae | 3 | 9.60 | 9.60 | 9.60 | 4.70 | 4.7 | 4.70 | 0.00 | 4.90 | 4.90 |
| 60 | Tra_gimenezi | 1 | 3.90 | 3.90 | 3.90 | -1.00 | -1 | -1.00 | 0.00 | 4.90 | 4.90 |
| 61 | Tra_orobios | 16 | 4.94 | 7.90 | 1.20 | 0.03 | 3 | -3.80 | 6.70 | 11.70 | 4.92 |
| 62 | Trc_mestrei | 2 | 9.90 | 9.90 | 9.90 | 5.00 | 5 | 5.00 | 0.00 | 4.90 | 4.90 |
| 63 | Tro_elongatus | 10 | 13.60 | 15.40 | 12.20 | 9.12 | 11 | 7.70 | 3.20 | 7.70 | 4.48 |
| 64 | Tro_espanoli | 1 | 13.40 | 13.40 | 13.40 | 8.90 | 8.9 | 8.90 | 0.00 | 4.50 | 4.50 |
| 65 | Tro_ferreri | 103 | 14.51 | 16.20 | 13.90 | 10.06 | 11.7 | 9.40 | 2.30 | 6.80 | 4.45 |
| 66 | Tro_fonti | 21 | 8.16 | 10.90 | 4.20 | 3.40 | 6.2 | -0.60 | 6.70 | 11.50 | 4.76 |
| 67 | Tro_hustachei | 3 | 8.33 | 9.10 | 7.50 | 3.63 | 4.4 | 2.80 | 1.60 | 6.30 | 4.70 |
| 68 | Tro_jacasi | 2 | 12.45 | 12.50 | 12.40 | 7.95 | 8 | 7.90 | 0.10 | 4.60 | 4.50 |
| 69 | Tro_kiesenwetteri | 11 | 13.64 | 15.70 | 11.50 | 9.11 | 11.1 | 6.90 | 4.20 | 8.80 | 4.53 |
| 70 | Tro_ludovici | 1 | 12.20 | 12.20 | 12.20 | 7.40 | 7.4 | 7.40 | 0.00 | 4.80 | 4.80 |
| 71 | Tro_olerdolai | 1 | 15.80 | 15.80 | 15.80 | 11.30 | 11.3 | 11.30 | 0.00 | 4.50 | 4.50 |
| 72 | Tro_orcinus | 12 | 12.79 | 13.90 | 10.80 | 8.31 | 9.4 | 6.30 | 3.10 | 7.60 | 4.48 |
| 73 | Tro_quadricollis | 2 | 8.70 | 9.80 | 7.60 | 3.95 | 5.1 | 2.80 | 2.20 | 7.00 | 4.75 |
| 74 | Tro_rovirai | 1 | 8.20 | 8.20 | 8.20 | 3.30 | 3.3 | 3.30 | 0.00 | 4.90 | 4.90 |
| 75 | Tro_schibii | 1 | 12.10 | 12.10 | 12.10 | 7.60 | 7.6 | 7.60 | 0.00 | 4.50 | 4.50 |
| 76 | Tro_senenti | 2 | 9.95 | 10.40 | 9.50 | 4.60 | 4.8 | 4.40 | 0.90 | 6.00 | 5.35 |
| 77 | Tro_subilsi | 2 | 9.45 | 12.70 | 6.20 | 4.75 | 8 | 1.50 | 6.50 | 11.20 | 4.70 |
| 78 | Tro_vinyasi | 3 | 10.73 | 12.20 | 9.80 | 6.00 | 7.4 | 5.10 | 2.40 | 7.10 | 4.73 |
| 79 | Trp_aubryi | 1 | 9.00 | 9.00 | 9.00 | 4.10 | 4.1 | 4.10 | 0.00 | 4.90 | 4.90 |
| 80 | Trp_bedeli | 1 | 13.50 | 13.50 | 13.50 | 8.60 | 8.6 | 8.60 | 0.00 | 4.90 | 4.90 |
| 81 | Trp_gavoyi | 2 | 10.65 | 10.90 | 10.40 | 5.80 | 6.1 | 5.50 | 0.50 | 5.40 | 4.85 |
| 82 | Trp_ludovici | 1 | 9.90 | 9.90 | 9.90 | 5.00 | 5 | 5.00 | 0.00 | 4.90 | 4.90 |
| 83 | Trp_nsspecie | 1 | 11.50 | 11.50 | 11.50 | 6.60 | 6.6 | 6.60 | 0.00 | 4.90 | 4.90 |
